# Effects of simulated space conditions on CD4+ T cells: a multi modal analysis

**DOI:** 10.3389/fimmu.2024.1443936

**Published:** 2024-09-02

**Authors:** Silvana Miranda, Randy Vermeesen, Ann Janssen, Emil Rehnberg, Emre Etlioglu, Sarah Baatout, Kevin Tabury, Bjorn Baselet

**Affiliations:** ^1^ Radiobiology Unit, Institute for Nuclear Medical Applications, Belgian Nuclear Research Centre SCK CEN, Mol, Belgium; ^2^ Department of Biotechnology, Faculty of Bioscience Engineering, Ghent University, Ghent, Belgium; ^3^ Department of Biomedical Engineering, College of Engineering and Computing, University of South Carolina, Columbia, SC, United States

**Keywords:** immune system, simulated microgravity, ionizing radiation, iron ions, space environment, cortisol, CD4 + T cells

## Abstract

**Introduction:**

The immune system is an intricate network of cellular components that safeguards against pathogens and aberrant cells, with CD4+ T cells playing a central role in this process. Human space travel presents unique health challenges, such as heavy ion ionizing radiation, microgravity, and psychological stress, which can collectively impede immune function. The aim of this research was to examine the consequences of simulated space stressors on CD4+ T cell activation, cytokine production, and gene expression.

**Methods:**

CD4+ T cells were obtained from healthy individuals and subjected to Fe ion particle radiation, Photon irradiation, simulated microgravity, and hydrocortisone, either individually or in different combinations. Cytokine levels for Th1 and Th2 cells were determined using multiplex Luminex assays, and RNA sequencing was used to investigate gene expression patterns and identify essential genes and pathways impacted by these stressors.

**Results:**

Simulated microgravity exposure resulted in an apparent Th1 to Th2 shift, evidenced on the level of cytokine secretion as well as altered gene expression. RNA sequencing analysis showed that several gene pathways were altered, particularly in response to Fe ions irradiation and simulated microgravity exposures. Individually, each space stressor caused differential gene expression, while the combination of stressors revealed complex interactions.

**Discussion:**

The research findings underscore the substantial influence of the space exposome on immune function, particularly in the regulation of T cell responses. Future work should focus expanding the limited knowledge in this field. Comprehending these modifications will be essential for devising effective strategies to safeguard the health of astronauts during extended space missions.

**Conclusion:**

The effects of simulated space stressors on CD4+ T cell function are substantial, implying that space travel poses a potential threat to immune health. Additional research is necessary to investigate the intricate relationship between space stressors and to develop effective countermeasures to mitigate these consequences.

## 1 Introduction

The immune system is comprised of numerous cellular components that protect the body against harmful pathogens and abnormal cells. Among these components, CD4 + T cells play a pivotal role in the immune response (1, 2). Named after the CD4 + glycoprotein, which is expressed on the cell surface and functions as a coreceptor for antigen recognition, CD4 + T cells are also referred to as T helper cells because their primary function is to support other immune cells (3). Specifically, they assist in the immune response by recognizing antigens presented by antigen-presenting cells such as dendritic cells and macrophages. When they identify an antigen and receive co-stimulatory signals, CD4 + T cells become activated and perform their functions (4, 5). Once activated, these cells release cytokines. The specific type of cytokine released is determined by the nature of activation, which in turn dictates the type of immune response that follows (6). Consequently, the type of CD4+ T cells produced depends on the cytokine environment and the nature of the antigen that initiates activation, which can be broadly categorized into four main subtypes (6).

**Figure 1:**
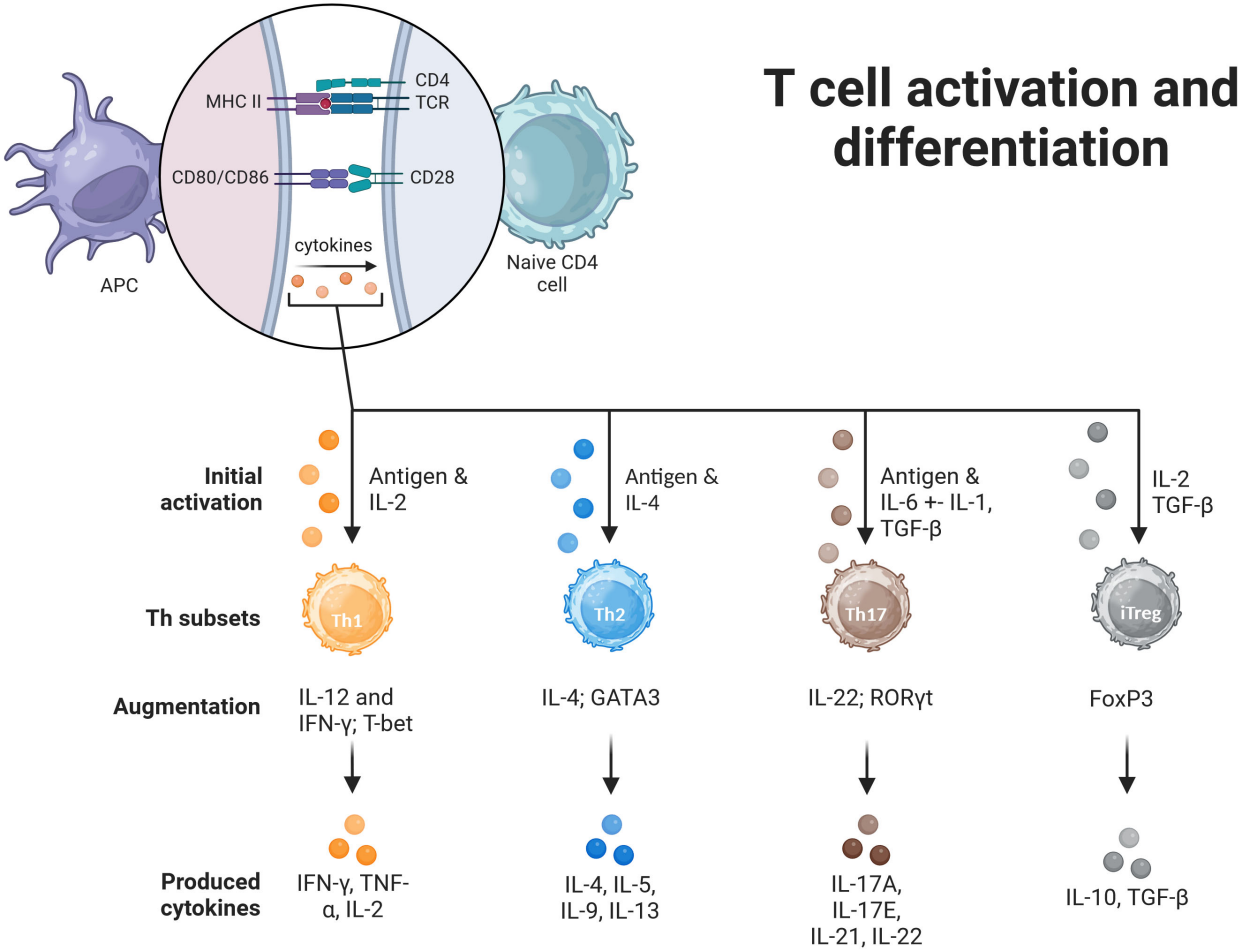
Induction of differentiation of Th cells. For the activation of T cells, a double signal process is required. First, the binding of the T-cell receptor (TCR) to the peptide-MHCII complex on the antigen-presenting cell (APC) surface. Binding of the T cell co-receptor CD28 to CD80/CD86 on the APC surface is the second signal required for complete activation. The fate of the differentiation depends on the combination of the antigen that initiated the response. Th1 cells are activated in response to IL-2, that induces T-bet expression and a subsequent Th1 cytokine profile. Th2 cells are induced by IL-4, express GATA3 and start Th2 cytokine profile. Th17 cells are induced by IL-6 and IL-1 to express Retinol-Related Orphan Receptor (ROR)γt and start producing IL-17, IL-21, IL-22 cytokines. The augmentation phase is promoted by specific sets of cytokines, further inducing Th subtype expansion, in reponse to the specific environment. Induced regulatory T cells (iTregs), are naïve T cells that express forkhead box P3 (FoxP3), and assume immunosuppressive functions. Image created in BioRender.

Cellular immune response against intracellular pathogens, such as bacteria, viruses, and parasites, is mainly mediated by T helper type 1 (Th1) cells. These cells develop from naive CD4+ T cells that have been stimulated with interleukin-12 and interferon-gamma. They release cytokines that aid in T cell development and differentiation, including IL-2 and tumor necrosis factor (TNF-γ), which activate macrophages and increase their capacity for microbicidal response (7). Th1 cells contribute to the development of a cell-mediated immune response that is crucial for killing infected host cells, eliminating intracellular pathogens, and participating in delayed-type hypersensitive reactions. Additionally, they assist B cells in producing opsonizing antibodies that can target pathogens for phagocytosis with a focus on IgG class switching (8). Th2 cells are linked to the humoral aspect of immunity, which primarily involves defense against extracellular parasites such as helminths. These cells also play a significant role in allergic responses. They differentiate from naïve CD4+ T cells under the influence of cytokines such as IL-4. Th2 cells produce various cytokines including IL-4, IL-5, IL-9, and IL-13. IL-4 is critical for B-cell class switching to generate IgE antibodies, which are involved in both allergic and antiparasitic responses. IL-5 promotes eosinophil growth and differentiation whereas IL-13 is involved in mucosal immunity and goblet cell production. Th2 cells play a central role in the development and progression of allergic diseases (7, 9). Regulatory T cells (Tregs) are a specialized subset of CD4+ T cells that play vital roles in preserving immune tolerance and preventing autoimmune diseases. These cells control the intensity of immune reactions by regulating the activity of CD4+ and CD8+ effector T cells, thereby preventing overactive responses that could cause tissue damage. Tregs produce anti-inflammatory cytokines, such as transforming growth factor-beta, interleukin-10, and IL-35, which directly suppress the activity of other immune cells. Additionally, Tregs can interact directly with other immune cells to fine-tune their immune response (10). Th17 CD4+ T cells are involved in antibacterial and antifungal responses. The name is derived from the signature cytokine secreted by these cells, IL-17. They are important for the recruitment of neutrophils to infection sites. IL-17 is also important for the amplification of the local immune response (7, 11). **Figure 1** aims to visually represent the activation, differentiation and expansion process for each Th subtype, highlighting the main cytokines needed to promote Th subtype differentiation and the panel of cytokines produced by each active subtype (7, 12).

Human space travel is currently in a transformative phase, with missions planned to travel greater distances and durations than ever before. However, as the potential for space travel continues to increase, significant health risks must be addressed. These health risks arise from the unique characteristics of the space environment, which poses several hazards to astronauts (13, 14). The space environment is characterized by factors that can be detrimental to human health. Exposure to ionizing radiation (IR) during space travel can lead to various health consequences, including an increased risk of cancer, DNA and cell damage, and impaired immune systems (15). Prolonged exposure to microgravity can result in detrimental changes in several human systems, such as the cardiovascular and musculoskeletal systems (16, 17). Furthermore, increased distance from Earth, confinement, isolation, immobilization, and lack of social interaction experienced by astronauts in space can lead to increased stress hormone levels that can also impact the homeostasis of biological systems (18).

Immune dysfunction associated with spaceflight has been described across several astronaut crews and in different space exploration contexts: either short- or long-duration missions, low earth orbit (LEO) missions, or (limited) beyond LEO missions (19). Microgravity is a prominent feature of the space environment that has profound effects on human physiology. Research has indicated that microgravity conditions can alter cellular behavior and immune system function. Studies have found that a lack of gravitational force in space can lead to immunosuppression, characterized by a reduction in T cell activation and proliferation (20). Microgravity affects the expression of genes crucial for T cell function. For instance, genes responsible for cytokine production, signaling pathways, and cellular stress responses are differentially regulated under these conditions (21). Ground-based simulations, such as clinostats and random positioning machines, mimic the effects of microgravity and have demonstrated similar outcomes, including changes in the cytoskeleton of T cells, which may disrupt cell-cell interactions and signaling required for proper immune function (22). Space travel exposes astronauts to IR from galactic cosmic rays and solar particle events, including high-energy protons and heavy ions. This type of radiation can penetrate biological tissues and cause direct damage to DNA as well as secondary effects, such as the generation of reactive oxygen species (23, 24). Consequently, T cell functionality can be impaired, affecting their ability to recognize antigens, proliferate, and secrete essential cytokines necessary for mounting an immune response. Long-term risks include an increased potential for radiation-induced cancer and persistent detrimental alterations to the immune system, which are of significant concern for deep space missions (25). Extended spaceflight also causes astronauts to experience psychological stress, that may arise from factors such as isolation and confinement, which can lead to elevated levels of stress hormones, including cortisol (26). Cortisol is known for its immunomodulatory effects, and its chronic elevation can lead to immunosuppression (27). T cells, particularly CD4+ T cells, are susceptible to the effects of cortisol, and studies have indicated that high levels of cortisol can inhibit T cell proliferation and cytokine production (28). The stress experienced during space missions might modulate T cell responses, compromising immune defense and potentially increasing the risk of infections and delayed hypersensitivity reactions (29). Therefore, monitoring and mitigating stress-induced immunological changes are crucial for maintaining astronaut health during space missions. Together, these factors contribute to the complex immunological challenges faced by astronauts. Understanding the cumulative impacts of microgravity, radiation, and stress on T cells is essential for designing effective countermeasures to protect the health of astronauts during future long-duration spaceflights (30).

The goal of this study was to isolate CD4+ T cells from the blood of healthy volunteers and expose them to simulated space stressors (see **Table 1** for a list of the conditions) in a single and combined manner to investigate their effects on T cell activation, gene expression, and cytokine production. Cytokine levels for Th1, Th2 cell populations were measured using multiplex Luminex assays to assess functional changes in T cell subsets under simulated space conditions. Furthermore, this study aimed to analyze gene expression patterns using RNA-seq to identify key genes and pathways involved in T cell activation and the immune response that are significantly altered in response to simulated space conditions. Overall, this study aimed to provide a comprehensive understanding of the effects of simulated space conditions on CD4+ T cells and their role in the immune response.

**Table 1:** Listing of the conditions in the experimental setup.

Radiation Exposure	Concentration of hydrocortisone (HC)	Gravity
0 Gy	0 µM	Earth 1 g (1_g)
1 Gy X-Rays (Photons)	1 µM	Micro ~0.01 g (s_µ_g)
1 Gy Iron ions (Fe)		

## 2 Materials and methods

### 2.1 Blood collection, CD4+ T cell extraction and cryopreservation

21 healthy male volunteers who met the inclusion criteria (see **Supplementary Materials**) were selected for this study. Blood samples were collected in 9mL lithium heparin tubes (Vacuette, Greiner Bio-One GmbH, Kremsmunster, Austria). Hematological analysis was performed using a Sysmex XS-800i automated hematology analyzer (Sysmex Corporation, Kobe, Japan). The instrument was calibrated and operated according to the manufacturer’s instructions. CD4+ T cells were isolated by negative selection with RosetteSep Human CD4+ T Cell Isolation Kits (StemCell Technologies, Inc. Vancouver, BC, Canada) and according to the manufacturer’s instructions. The isolated CD4+ T cells were cryopreserved in heat inactivated fetal bovine serum (HI FBS) with 10% DMSO for later use in the experimental procedures. Flow cytometry analysis was performed to confirm the purity of isolated CD4+ T cells using a gating strategy based on the expression of CD3 and CD4 markers. (see **Supplementary Materials**).

**Figure 2:**
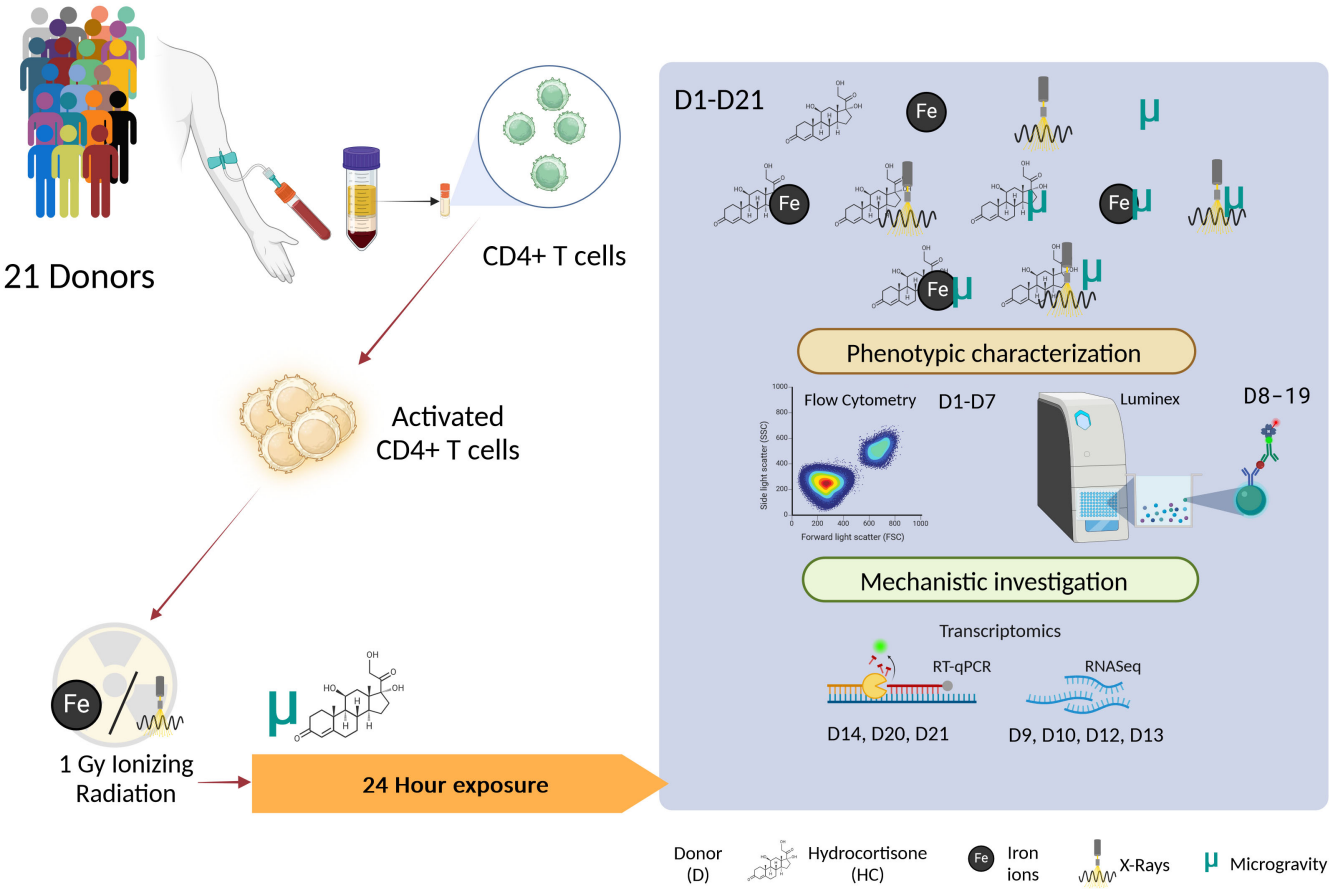
Schematic overview of the experimental design. The different combinations of the exposures to simulated space conditions are represented in the highlighted box. D, Donor; HC, Hydrocortisone; Fe, Iron ion irradiation; ELISA, Enzyme Linked Immunosorbent Assay. Image created with BioRender.

Upon thawing, cryopreserved CD4+ T cells were stimulated with an anti-CD3/CD28 stimulation cocktail from StemCell Technologies at a concentration of 1 μg/mL in complete T cell expansion medium (StemCell Technologies, Inc. Vancouver, BC, Canada). The cells were then divided into experimental groups. Stimulated CD4+ T cells were incubated at 37°C with 5% CO2 for 24 h to allow for maximum activation and cytokine secretion, without compromising on cell viability. **Figure 2** provides a schematic overview of the experimental design and **Table 2** provides the list of donors and the assays performed.

**Table 2:** Donor list and utilization for each assay.

Donor	Assay			
	Flow cytometry	Luminex	RT-qPCR	RNASeq
D1	✔			
D2	✔			
D3	✔			
D4	✔			
D5	✔			
D6	✔			
D7	✔			
D8		✔		
D9		✔		✔
D10		✔		✔
D11		✔		
D12		✔		✔
D13		✔		✔
D14		✔	✔	
D15		✔		
D16		✔		
D17		✔		
D18		✔		
D19		✔		
D20			✔	
D21			✔	

### 2.2 Microgravity simulation

The simulated microgravity conditions were created using an RPM 2.0 (Yuri Gravity). Air bubbles are a cause of concern regarding microgravity experiments due to the impact they may have on the cells (31). The vial caps were sealed using a polymer (SYLGARD 184 Silicone Elastomer, Dow, 01673921), allowing airtight sealing of the vial. To account for the lack of gas exchange during the experimental setup time, the media were previously allowed to reach gas equilibrium in an incubator (37°C with 5% CO2 in air atmosphere). The cells were exposed to simulated microgravity for 24 h, intended to reflect initial cellular adaptations to microgravity, representing an acute phase response which is critical for understanding early cellular behavior in spaceflight. This timeframe aligns with the goal to first elucidate the immediate cellular mechanisms before extending to long-duration effects in future studies.

### 2.3 Ionizing radiation exposure

Photon irradiation was performed at the Laboratory of Nuclear Calibration of the Belgian Nuclear Research Center (SCK CEN) in Mol, Belgium. For photon irradiation, cell culture flasks were placed on a Plexiglass plate in a horizontal position, irradiated from the top at a distance of 50 cm, and irradiated with X-rays (Irradiator Xstrahl 320 kV H-250, air kerma (Kair) 1.00 Gy with uncertainty on the conventional value (k = 2) of 0.06 Gy). Irradiation was performed at the Laboratory of Nuclear Calibration of the Belgian Nuclear Research Center (SCK CEN) in Mol, Belgium.

Iron ion irradiation was performed at the Grand Accelerateur de Ions de Lourdes (GANIL) in Caen, France. Owning to the requirement for the samples to be placed vertically to the beam line window, fluoro-ethyl polymer (FEP) cell culture bags (PL07-2G, Origen Biomedical, Austin, TX, USA) were used to irradiate CD4+ T cells to obtain an even distribution of the dose For both irradiations, the cells were irradiated with a total dose of 1 Gy.

For both irradiations, the cells were irradiated with a total dose of 1 Gy. This approach is particularly relevant given the limited data on the specific relative biological effectiveness (RBE) values for immune cells, which are still not fully characterized in the literature. Research suggests that RBE can vary significantly depending on the biological endpoint, the type of cells, and the radiation environment (25, 32). By comparing the effects of these two radiation types at the same dose, this study aims to elucidate differential pathways of cellular response that could inform future radiobiological models, particularly in the context of mixed-field environments encountered in space travel. This method allows for a clear, direct comparison of the cellular impacts induced by each radiation type, providing data that can guide more detailed investigations into dose and RBE relationships in immune cells.

### 2.4 Stress hormone exposure

Hydrocortisone (HC) was used as a synthetic analog to cortisol. A stock solution of 1 mg/mL (2.76 mM) of HC (Sigma-Aldrich^®^, H0888) was prepared in dimethyl-sulfide (DMSO). This stock solution was dissolved in DMSO to obtain a concentration of 100 µM. The HC solution was 1/100 diluted in cell culture media to obtain a final working HC concentration of 1 µM. DMSO vehicle was added to the conditions not exposed to HC. The cells in the HC treated groups were exposed to the compound for 24 hours.

### 2.5 Flow cytometry

CD4+ T cells of the flow cytometry group (see **Table 2**) were treated with Brefeldin A for protein transport inhibition (32), (BioLegend, San Diego, CA, USA) according to the manufacturer’s instructions (briefly, a 5mg/mL stock solution was used for a final concentration in the cells of 5µg/mL). After the 24h exposure to simulated space conditions, cells were stained with an optimized multicolor immunofluorescence panel (OMIP008), (33), described in **Table 3**, from BioLegend (BioLegend, San Diego, CA, USA), according to the manufacturer protocol.

**Table 3:** OMIP008 panel for CD4+ T cell staining.

Specificity	Clone	Fluorochrome	Purpose
IL-2	MQ1-17H12	Brilliant Violet 711	Function
IL-4	8D4-8	Brilliant Violet 421	
IL-10	JES3-9D7	PE	
IFN-γ	B27	APC	
TNF-α	Mab11	PE/Cy7	
CD3	UCHT1	PerCP/Cy5.5	Lineage
CD4	RPA-T4	FITC	
CD8	3B5	Brilliant Violet 510	
CD14	M5E2	APC/Fire750	Dump
CD19	HIB19	APC/Fire750	
Dead cells	–	Zombie NIR	

PE, Phycoerythrin; APC, Allophycocyanin; PE-Cy7, Phycoerythrin conjugated to Cy7; PerCP-Cy5.5, Peridinin-chlorophyll-protein (PerCP complex) with Cy5.5 fluorophore; FITC, Fluorescein isothiocyanate; Zombie NIR, A near-infrared dye viability marker.

### 2.6 Luminex assay

In this study, the Luminex Enzyme Linked Immunosorbent Assay (ELISA) was used to measure the levels of multiple analytes in cell samples. Human XL Cytokine Luminex^®^ Kit Performance Assay (R&D BioTechne) was designed for the cytokines IFN-γ, TNF-α, IL-2, IL-4, IL-5, IL-13, and IL-10. Assays were performed according to the manufacturer’s instructions. Samples were run on a MAGPIX instrument (Luminex, Merck) and analyzed with MILLIPEX Analyst standard version 5.1 (Merck). The output was further analyzed using Belysa^®^ Immunoassay Curve Fitting Software (Merck, Rahway, NJ, USA).

### 2.7 Quantitative real-time polymerase chain reaction

Cell pellets were lysed using RLT plus buffer (Qiagen, Hilden, Germany) containing 1:100 β-mercaptoethanol. The RNeasy Plus Mini Kit (Qiagen) was used to extract total RNA following the manufacturer’s protocol. The purity and concentration of the isolated RNA were determined using NanoDrop™ 2000/2000 (Thermo Scientific™, Waltham, MA, USA). Reverse transcription of RNA into cDNA was performed using GoScript™ Reverse Transcriptase ((Promega, Alexandria, Australia) for each sample.

RT-qPCR was performed to assess gene expression for the CD4+ T cells Th subtypes Th1, Th2, was performed using the Rotor-Gene Q instrument (Qiagen, Hilden, Germany). TaqMan Gene Expression Assays (Applied Biosystems, USA) for TBX21 (Assay ID: [Assay ID]), GATA3 (Assay ID: [Assay ID]), FOXP3 (Assay ID: [Assay ID]), 18S rRNA (Assay ID: [Assay ID]), or HPRT1 (Assay ID: [Assay ID]) were used according to the manufacturer instructions. The thermal cycling conditions were as follows: initial denaturation at 95°C for 10 minutes, followed by 40 cycles of 95°C for 15 seconds, and 60°C for 1 minute. Fluorescence data was collected at the end of each annealing/extension phase, and threshold cycle (Ct) values were calculated using the Rotor-Gene Q software (version 2.3.1). Relative expression levels were determined using the 2^-ΔΔCt method, with 18S rRNA and HPRT1 serving as internal reference genes. Negative controls (no-template controls were included in each run to ensure the validity of the results.

### 2.8 RNA sequencing

#### 2.8.1 Sample preparation

Total RNA was extracted from isolated CD4^+^ T cells using the RNeasy Plus Mini Kit (Qiagen, Germany), according to the manufacturer’s protocol. RNA integrity was assessed using the Agilent RNA 6000 Pico Kit and 2100 BioAnalyzer (Agilent Technologies Inc., Santa Clara, CA, USA). For all samples, the RIN values met the requirements for high-quality RNA-sequencing analysis.

#### 2.8.2 Library preparation and sequencing

Messenger RNA was purified from total RNA using poly-T oligo-attached magnetic beads. After fragmentation, the first-strand cDNA was synthesized using random hexamer primers, followed by second strand cDNA synthesis using either dUTP for the directional library or dTTP for the non-directional library. The non-directional library was prepared after end repair, A-tailing, adapter ligation, size selection, amplification, and purification. The directional library was prepared after end repair, A-tailing, adapter ligation, size selection, USER enzyme digestion, amplification, and purification. The library was checked with Qubit and real-time polymerase chain reaction (RT-PCR) for quantification and a bioanalyzer for size distribution detection. Quantified libraries were pooled and sequenced on Illumina platforms according to the effective library concentration and data amount.

#### 2.8.3 RNA-seq data analysis

Quality control and preprocessing of the RNA-seq data was performed with the Nextflow pipeline nf-core/rnaseq version 3.12.0 (34) using the default values except for: i) Ensembl GRCh38 release 109 was used as the reference genome, ii) salmon quant was run with the “–gcBias” parameter.

Differential gene expression analysis was performed with the Nextflow pipeline nf-core/differential abundance version 1.4.0 (34) with the default values. Gene Set Enrichment Analysis (GSEA) was performed using the Molecular Signatures Database (MSigDB) Hallmarks collection (gene signatures representing key biological processes and pathways). These collections encompass a wide range of biological pathways, regulatory motifs, functional annotations, and immunologic signatures, providing comprehensive coverage for analyzing gene expression data in this study.

### 2.9 Data visualization and statistical analysis

Visualization and statistical analysis for population characteristics, FC, Luminex, and RT-qPCR data was performed with Rstudio version 4.3.1. Detailed information regarding the statistical tests and additional visualization can be found in **Supplementary Materials**.

### 2.10 Ethical considerations and approval

The present study was conducted in strict accordance with ethical principles outlined in the Declaration of Helsinki and was approved by the UZLeuven Ethics Committee, study number: S65343. All participants provided written informed consent prior to their inclusion in the study, after being comprehensively briefed on the purpose, procedures, and potential risks involved. Confidentiality of participant information was rigorously maintained throughout the study duration, with data anonymized and stored securely. Participation in the study was entirely voluntary, and participants were informed of their right to withdraw at any stage without repercussion. Special consideration was given to vulnerable populations, and additional safeguards were implemented to ensure their welfare.

## 3 Results

### 3.1 Population sample characterization

The average age of the population under study was 37.52 years, with a median age of 36 years. At the moment of sample collection for CD4 + T cell extraction, a sample was collected for blood analysis using the Sysmex XS-800i automated hematology analyzer via flow cytometry. White blood cell (WBC) counts, lymphocyte percentage were measured. WBC count ranged from 3.7 to 7.97 x 10^3/μL, with a mean of 5.92 x 10^3/μL ± 1.13 x 10^3/μL, while the lymphocyte percentage varied between 17.9% and 46.5%, with a mean of 30.45% ± 6.85%. These data provide essential insights into the demographic and hematological characteristics of the population, which are crucial for our subsequent data analysis.

Correlation analysis was performed to investigate any interactions between the population characteristics that could influence CD4 + T cell behavior. No significant correlations were found between age of the volunteers and their WBC count or lymphocyte population. Additional details of the statistical analysis of the population characteristics can be found in **Supplementary Materials**.

### 3.2 Exposure to simulated space conditions did not impact CD4+ T cell viability

**Figure 3:**
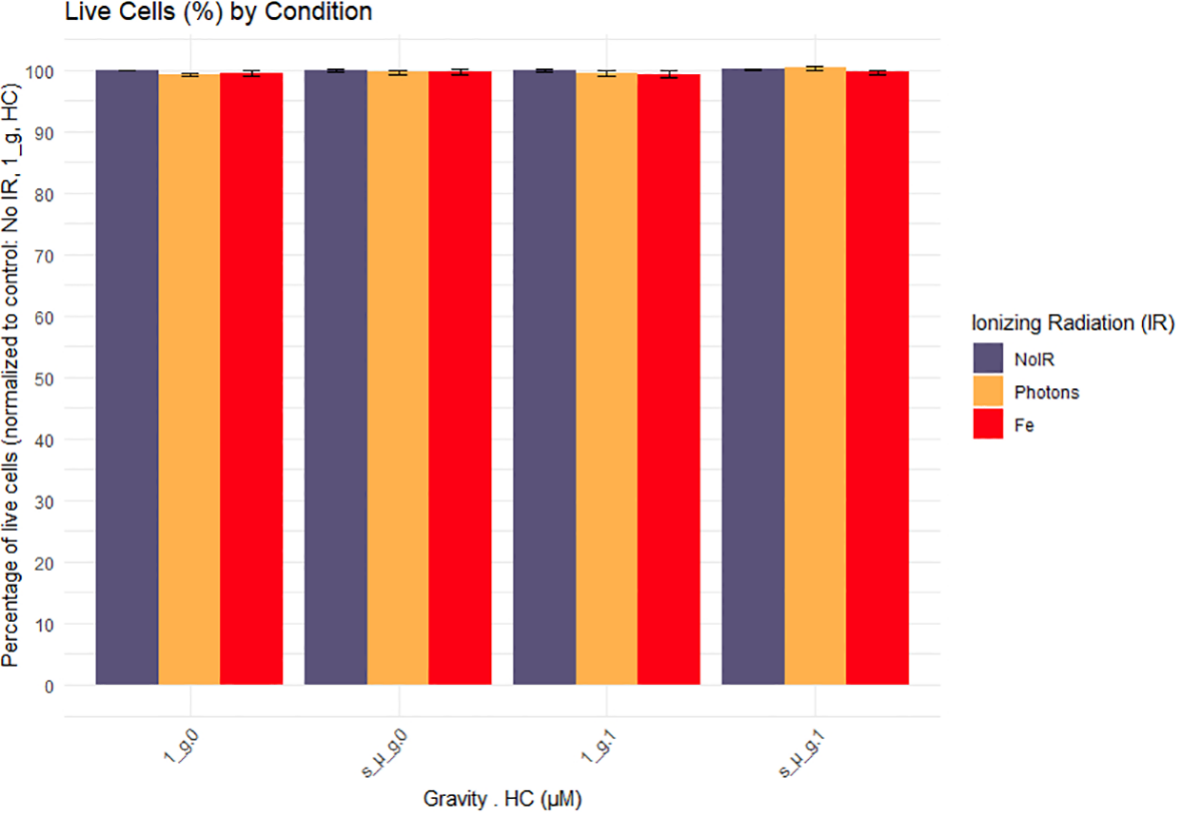
Cell viability 24h after exposure to simulated space conditions. 1_g, normal gravity; s_µ_g, Simulated microgravity; HC, Hydrocortisone. Values represented are normalized to the control (No Ionizing radiation (IR), 0 µM HC, 1_g) and the errors bars the SEM. The x axis shows the gravity levels combined with HC. comparing the distribution of the normalized cytokine levels across different IR exposures [No IR, Photons, Fe (ions)], represented by different fill colors. Data was obtained from 6 donors and analyzed with RStudio v4.3.1.

CD4 + T cells from 6 donors were used for flow cytometry analysis. Brefeldin A was added to the cells at the start of the experiment in order to block cytokines secretion for later intracellular staining procedures. Outliers were identified and removed from the dataset using the interquartile range (IQR) method. The percentage of live cells was obtained to evaluate the effects of the exposure to IR, simulated microgravity and HC, normalized to the control condition (the counts of positive-staining death cells over the total of the events within the CD4+ T cell staining population). We observed that the simulated space conditions did not induce changes in the viability of the cells at the 24h time point evaluated (**Figure 3**).

For the duration of the experiment, cells were treated with Brefeldin A, disrupting their protein trafficking (35). Cells were then stained for intracellular cytokines to evaluate the production of the characteristic Th cytokines (IFN-γ, TNF-α, IL-2, IL-4, and IL-10). A considerable variability between donors, was observed and results from this analysis were inconclusive (for more details, see **Supplementary Materials**).

Cytokine measurements in supernatant serve as the primary method for assessing the impact of exposure to stress factors on the CD4+ T cell phenotype. In the following section, the outcomes of the Luminex analyses are elucidated.

### 3.3 Evaluation of the effects of simulated space conditions on the cytokine levels shows different effects of IR, HC, and simulated microgravity exposure, and a complex interaction effect

#### 3.3.1 IFN-γ, TNF-α, and IL-2: Th1 response

**Figure 4:**
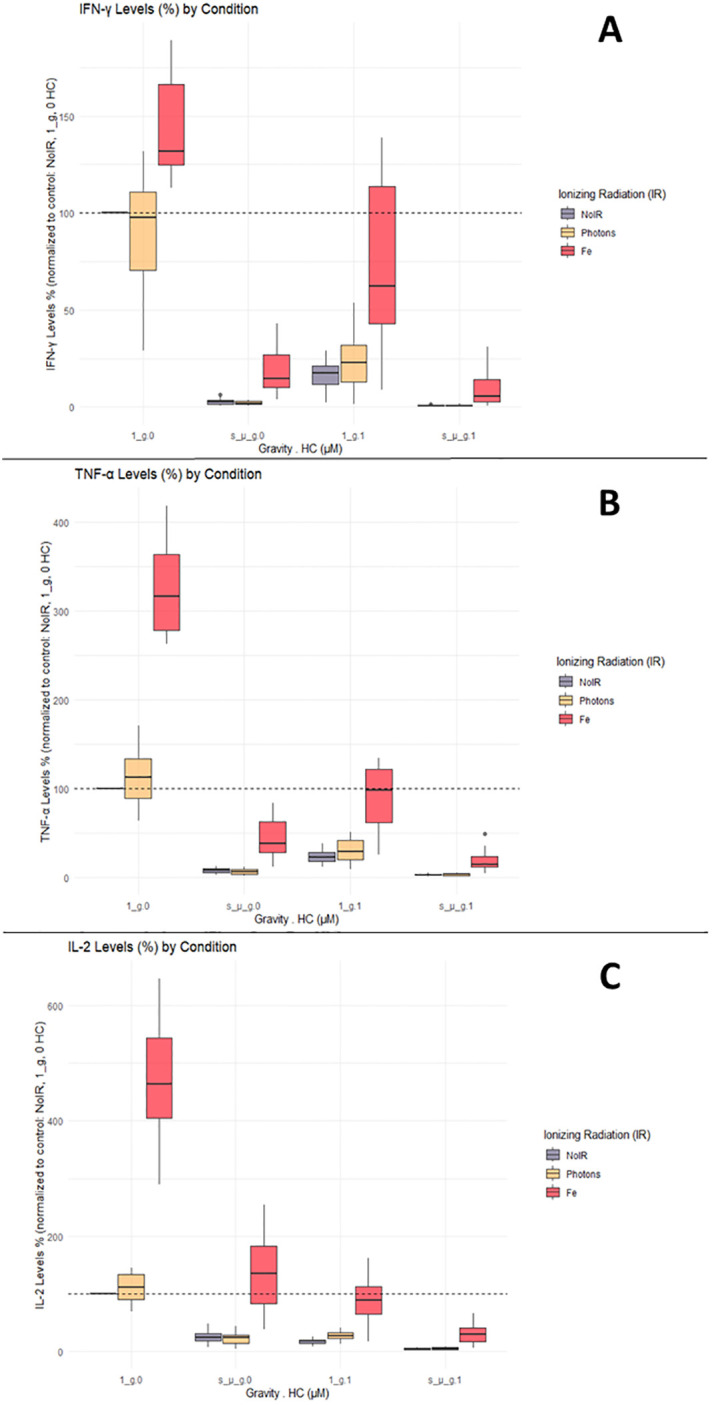
IFN-γ, TNF-α, and IL-2 cytokine levels. Luminex assay results analyzed with Belysa software (Merck). **(A)** IFN-γ; **(B)** TNF-α; **(C)** IL-2. 1_g, normal gravity; s_µ_g, Simulated microgravity; HC, Hydrocortisone. Values represented are normalized to the control [No Ionizing radiation (IR), 0 µM HC, 1_g]. Plot shows boxplot with median as center line. The bottom and top edges of the box represent the 1st quartile (Q1) and the 3rd quartile (Q3) of the data, respectively. The height of the box (the interquartile range or IQR) represents the middle 50% of the data. The whiskers represent 1.5 times the IQR. Points represent outliers. The x axis shows the gravity levels combined with HC. comparing the distribution of the normalized cytokine levels across different IR exposures [No IR, Photons, Fe (ions)], represented by different fill colors of the boxes. The horizontal dashed line is indicating the average value for the control condition. Data was obtained from 12 donors and analyzed with RStudio v4.3.1.

IFN-γ, TNF-α and IL-2 are important cytokines mediating Th1 responses (36). Luminex ELISA was performed on the supernatant obtained from the CD4+ T cells of 12 donors, exposed to the different combinations of simulated space conditions. IFN-γ, TNF-α, and IL-2 cytokine levels increased with exposure to IR alone, specifically Fe irradiation, where the values were up to 4 times higher compared to the normalized values for the control (**Figure 4C**, normal gravity and 0 µM HC (1_g.0) group). Photon irradiation also induced a discrete increase in cytokine levels compared with the control conditions. Simulated microgravity and HC exposure decreased the production of IFN-γ, TNF-α, and IL-2 compared to the control conditions (**Figures 4A–C**). Combined exposure to simulated microgravity and HC exhibited the greatest decrease in cytokine production. When radiation was combined with either simulated microgravity or HC, there was an apparent ‘recovery’ of the cytokine levels when compared to the exposure to simulated microgravity or HC alone. However, when the cells were exposed to all three simulated space stressors, the recovery effect was lost.

These results suggest that the effects of simulated space conditions on CD4+ T cells can modulate cytokine production, with IR increasing cytokine production, and simulated microgravity or HC decreasing cytokine production. If IR is combined with HC or simulated microgravity, an increase of the cytokine levels is seen (compared to HC or simulated microgravity alone). However, if both HC and simulated microgravity are present, this IR effect is no longer found.

#### 3.3.2 IL-4, IL-5, IL-13: Th2 response

**Figure 5:**
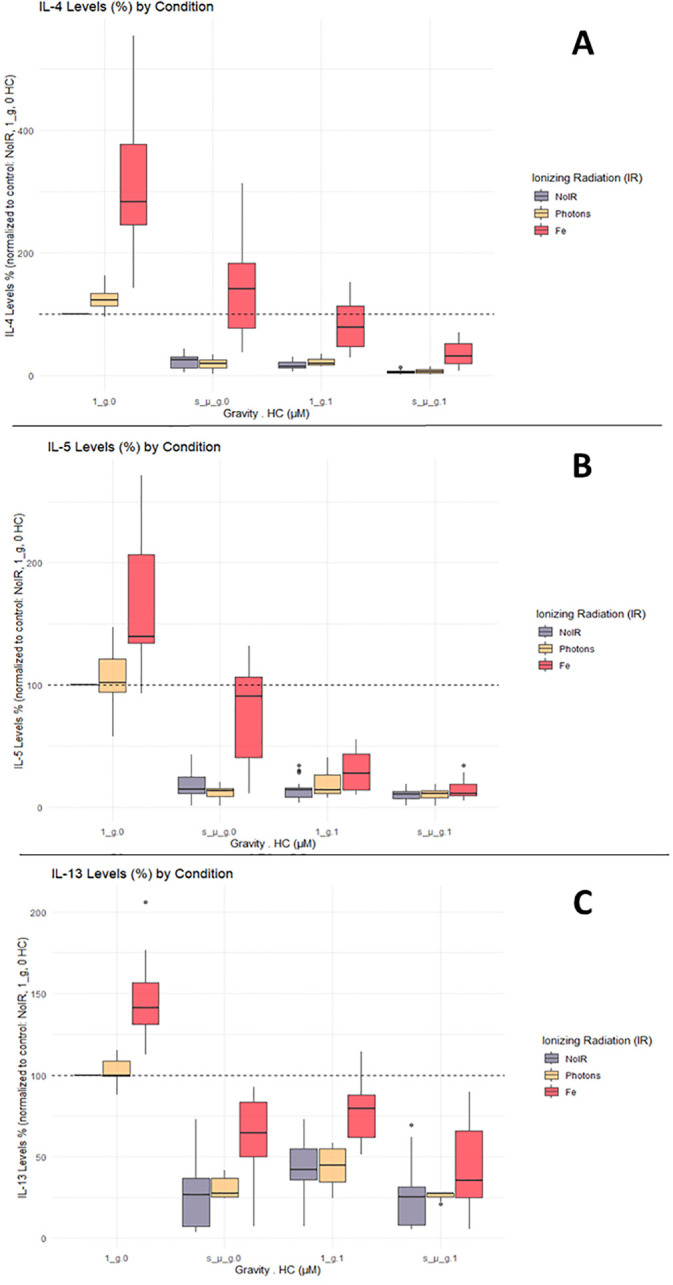
IL-4, IL-5, and IL-13 cytokine levels. Luminex assay results analyzed with Belysa software (Merck). **(A)** IL-4; **(B)** IL-5; **(C)** IL-13. 1_g, normal gravity; s_µ_g, Simulated microgravity; HC, Hydrocortisone. Values represented are normalized to the control [No Ionizing radiation (IR), 0 µM HC, 1_g]. Plot shows boxplot with median as center line. The bottom and top edges of the box represent the 1st quartile (Q1) and the 3rd quartile (Q3) of the data, respectively. The height of the box (the interquartile range or IQR) represents the middle 50% of the data. The whiskers represent 1.5 times the IQR. Points represent outliers. The x axis shows the gravity levels combined with HC. comparing the distribution of the normalized cytokine levels across different IR exposures [No IR, Photons, Fe (ions)], represented by different fill colors of the boxes. The horizontal dashed line is indicating the average value for the control condition. Data was obtained from 12 donors and analyzed with RStudio v4.3.1.

IL-4, IL-5 and IL-13 cytokines are characteristic of Th2 T cell subtypes. Data for IL-4, IL-5, and IL-13 measured in CD4+ T cells’ supernatant showed similar responses to the one observed for the IFN-γ, TNF-α, and IL-2. Compared with the normalized control levels, exposure to IR alone increased cytokine levels. Compared to IFN-γ, TNF-α, and IL-2, the levels of IL-4, IL-5, and IL-13 were not as high in the Fe ion-exposed groups (**Figure 5**, Fe conditions in A, B, and C for the normal gravity and 0 µM HC (1_g.0) group). Simulated microgravity and HC exposure decreased the production of IL-4, IL-5, and IL-13 compared to the control conditions. The combination of simulated microgravity and HC led to the greatest decrease in cytokine production, similarly to the observations in **Figure 5**. When combined with IR, there was a partial recovery of IL-4, IL-5, and IL-13 cytokine levels compared with exposure to simulated microgravity or HC alone. Similar to IFN-γ, TNF-α, and IL-2, when the cells are exposed to IR and both HC and simulated microgravity, this IR effect is likely abrogated.

Overall, these findings suggest that simulated space conditions can significantly affect cytokine production by CD4+ T cells and that complex interactions between simulated space stressors affect CD4 + T cell responses. In order to better elucidate the nature of these interactions, statistical analysis for the Luminex data was performed.

#### 3.3.3 Assessing the interaction effects between simulated space conditions

In space, astronauts are simultaneously exposed to IR, microgravity, and elevated stress levels. However, little is known about the effects of each factor alone and whether there are any interactions (and what kind) between them. To investigate this, linear regression models were applied to the data to understand the interaction effects of simulated space stressors (more details regarding the models created and the goodness of fit can be found in the **Supplementary Materials**).

**Table 4** summarizes the statistically significant estimated effects for each predictor and the interactions. The models for all cytokines were significant with p values <0.0001.

**Table 4:** Estimated effects of factors on cytokine Levels, including linear regression models’ p-values.

Factor	IFN-γ	TNF-α	IL-2	IL-4	IL-5	IL-13
**Fe**	63.3 ± 5	206.7 ± 9	309.6 ± 12	216.1 ± 12	66.4 ± 7	54.6 ± 6
**s_µ_g**	-97.0 ± 9	-91.8 ± 7	-74.3 ± 10	-77.8 ± 10	-82.4 ± 6	-80.6 ± 5
**HC**	-82.6 ± 7	-76.4 ± 7	-81.7 ± 10	-83.6 ± 10	-83.8 ± 6	-63.9 ± 6
**Fe:s_µ_g**	-48.0 ± 12	-170.5 ± 12	-220.9 ± 18	-131.2 ± 18		
**Fe: HC**		-139.9 ± 12	-239.7 ± 18	-151.5 ± 17	-46.7 ± 10	-20.0 ± 9
**s_µ_g:HC**	81.1 ± 10	71.5 ± 19	60.6 ± 14	66.8 ± 14	76.3 ± 9	52.6 ± 9
**Fe:s_µ_g:HC**		123.7 ± 17	176.2 ± 25	95.7 ± 25		
**model *p value***	< 2.2e-16	< 2.2e-16	< 2.2e-16	< 2.2e-16	< 2.2e-16	< 2.2e-16
**Model adjusted R^2^**	0.7978	0.9073	0.8783	0.8351	0.8391	0.8457
**Model AIC**	1748	1761	1843	1858	1681	1326

It can be seen that the model confirmed the observations from the boxplots (**Figures 3**–**5**), indicating a statistically significant increase of the cytokine levels with Fe ions irradiation. Exposure to HC and simulated microgravity (s_µ_g) also shows a statistically significant impact on the cytokine levels, this time with a suppressive effect. The estimate values for the interaction between Fe ions irradiation and simulated microgravity showcase the impact that exposure to microgravity has on the IR effect. The same can be said for the interaction effects between Fe ions and HC. The positive estimate value observed for interaction between HC and simulated microgravity suggests that when the cells are exposed to both, their suppressive effects seem to amplify each other (positive interaction effect). This could be because they target different pathways in the immune system, and their combined actions leads to a more pronounced reduction in cytokine levels. When the three simulated space stressors are present, we can observe that the presence of HC and simulated microgravity impacts the stimulatory effect of Fe ions exposure, and that this interaction is significant for TNF-α, IL-2, and IL-4.

Analysis of variance (ANOVA) was performed in order to assess the overall model fit and individual predictor significance. **Table 5** summarizes the results of the linear regression analysis the ANOVA performed to assess the significance of the interactions.

**Table 5:** Results from the ANOVA performed for the linear regression models executed for each cytokine and for the grouped cytokines per Th subtype.

Factor	IFN-γ	TNF-α	IL-2	IL-4	IL-5	IL-13
**Fe**	1.10E-15	<2.2E-16	<2.2E-16	<2.2E-16	<2.2E-16	9.01E-16
**Gravity**	<2.2E-16	<2.2E-16	<2.2E-16	<2.2E-16	<2.2E-16	<2.2E-16
**HC**	<2.2E-16	<2.2E-16	<2.2E-16	<2.2E-16	<2.2E-16	<2.2E-16
**Fe: Gravity**	5.88E-08	<2.2E-16	<2.2E-16	1.236e-09		0.0182
**Fe: HC**		<2.2E-16	<2.2E-16	< 2.2E-16	4.96E-12	
**Gravity: HC**	<2.2E-16	<2.2E-16	<2.2E-16	<2.2E-16	<2.2E-16	2.211E-14
**Fe: Gravity : HC**		1.89E-12	9.73E-12	0.0003		
**Residuals (Df)**	176	178	173	174	176	141

The results indicated that the assumption that, for all the cytokines evaluated, irradiation with Fe ions causes an increase in the levels of each of the cytokine evaluated that is highly significant (p value < 0,00001). Simulated microgravity and HC exposure have a highly significant suppressive effect on cytokine levels, confirming the observations of **Figures 4**, **5**. The combined exposure to simulated microgravity and HC also showed to have a larger suppressive effect on cytokine levels that is also statistically significant for all cytokines. The alterations observed due to photon irradiation were not shown to be statistically significant for any given cytokine.

Overall, these results highlight that exposure to different simulated components of the space environment has different effects on the CD4+ T cell biology, as observed by the results of the cytokine measured in the cells supernatant. IR exposure with Fe ions has a cytokine stimulatory effect and exposure to HC and simulated microgravity has a suppressive effect that is potentiated when the latter are combined. Moreover, in combination with Fe ions, it is shown that there is a suppression of the stimulatory effects of the IR exposure.

#### 3.3.4 Cytokine levels show an apparent Th2 shift under simulated microgravity

**Figure 6:**
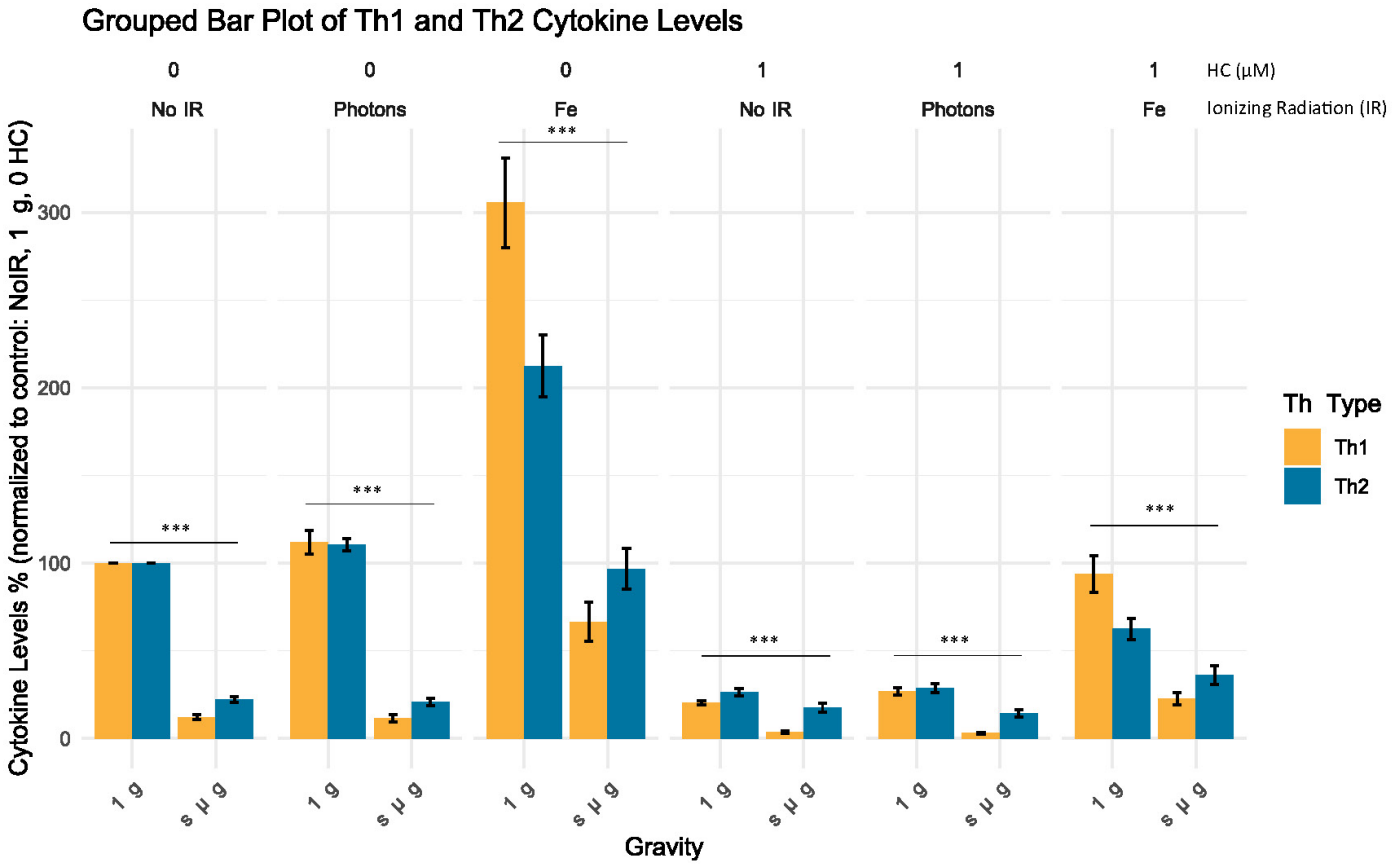
Grouped analysis of the cytokine level (%) for each Th subtype. Bar plot illustrates the mean cytokine levels (%) normalized to control for different Th groups (Th Type) across varying experimental conditions, with standard error of the mean (SEM) error bars. Statistical significance was determined using non-parametric Mann-Whitney U test for independent samples, with p-values indicating highly significant differences between the normal gravity experimental conditions (1 g) and the simulated microgravity (s µ g) experimental conditions (p < 2.2e-16, denoted by ***). No IR, No ionizing radiation; Photons, Photon irradiation; Fe, Iron ions irradiation; HC, Hydrocortisone. Data was obtained from 12 donors and analyzed with RStudio v4.3.1, annotations in the image added with Inkscape 1.3 (0e150ed6c4, 2023-07-21).

To assess whether exposure to simulated space stressors produced changes in the Th type of CD4^+^ T cells, a grouped analysis of the cytokines for the Th1 and Th2 subtypes was performed (see **Table 6**). The results show that for all the conditions where simulated microgravity was present, there was an apparent shift towards the Th2 profile that is statistically significant compared to all the normal gravity conditions, independent of the exposure to other simulated space stressors (**Figure 6**).

**Table 6:** Cytokine grouping according to Th subtype.

Th1 Cytokines	Th2 Cytokines
IFN-γ, TNF-α, and IL-2	IL-4, IL-5, IL-13

These findings add to the previous results, suggesting that simulated space conditions can alter the production of cytokines by CD4+ T cells and that simulated microgravity may lead to a shift towards a Th2 profile.

### 3.4 Gene expression analysis shows that microgravity induces an increased Th2 gene expression

**Figure 7:**
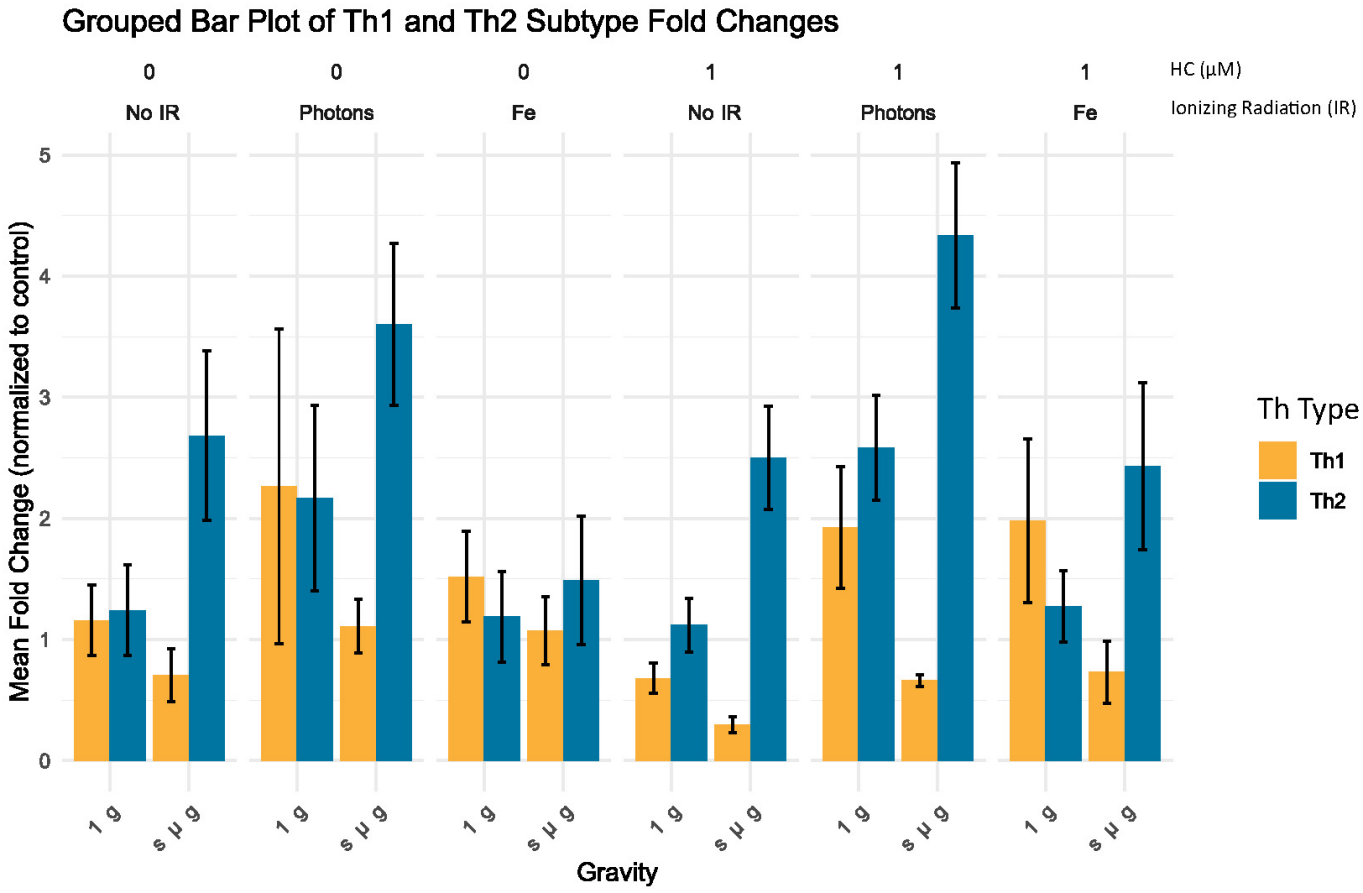
Grouped bar plot of Th subtype fold changes. Bar plot illustrates the mean fold change normalized to control (No IR, 1 g, 0 µM HC) for different Th groups (Th Type) across varying experimental conditions, with standard error of the mean (SEM) error bars. No IR, No ionizing radiation; Photons, Photon irradiation; Fe, Iron ions irradiation; HC, Hydrocortisone. Data was obtained from 3 donors and analyzed with Rstudio v4.3.1, annotations in the image added with Inkscape 1.3 (0e150ed6c4, 2023-07-21).

To further investigate the influence of simulated space conditions on the Th profile of CD4+ T cells, RT-qPCR was performed for a selected panel of genes. We selected specific genes that are well-established markers for different Th cell subtypes to understand the impact of various conditions on immune cell differentiation. **Table 7** summarizes the genes chosen for this analysis, their full names, the Th subtypes they are associated with, and their functional relevance in the immune response. Using the ΔΔCT method (39), we analyzed the fold changes in gene expression across different experimental groups. The grouped bar plot (**Figure 7**) illustrates the mean fold changes for each Th subset (Th1, Th2), stratified by Radiation, Gravity, and Cortisol levels. It can be observed that for the control condition (No IR, 1 g and 0 µM HC) there are no observable differences between the Th subtypes gene expression fold change. CD4+ t cells exposed to simulated microgravity alone, display an apparent shift towards Th2, showcased by the increased fold change of the GATA3 gene expression, respectively (**Figure 7**, No IR panel 0 µM HC). This shift seems to be consistent even in the presence of other simulated space conditions.

**Table 7:** RT-qPCR panel selection.

Gene	Full Name	Th Subtype	Function and Relevance
**TBX21**	T-Box Transcription Factor 21 (T-bet)	Th1	TBX21 encodes T-bet, a transcription factor crucial for Th1 cell differentiation. Th1 cells produce IFN-γ, which is involved in cell-mediated immunity (37).
**GATA3**	GATA Binding Protein 3	Th2	GATA3 is a transcription factor essential for Th2 cell differentiation. Th2 cells produce IL-4, IL-5, and IL-13, which are important in humoral immunity and allergic responses (38).

To assess the significance of the observed changes, we employed a linear mixed-effects model (LME) to account for donor variability. *Post-hoc* comparisons were made using estimated marginal means (EMM) and custom contrasts comparing Th1 with Th2 subsets under each experimental condition. The table below (**Table 8**) summarizes the significant contrasts.

**Table 8:** Table of significant contrasts obtained for the gene expression comparing Th1 to Th2.

Contrast	Radiation	Gravity	Cortisol	Estimate	SE	df	t-ratio	p-value	
Th1 vs Th2	NoIR	s_µ_g	0	-1.9773	0.461	106	-4.286	<.0001
Th1 vs Th2	Photons	s_µ_g	0	-2.4927	0.653	106	-3.82	0.0002
Th1 vs Th2	NoIR	s_µ_g	1	-2.2003	0.461	106	-4.769	<.0001
Th1 vs Th2	Photons	s_µ_g	1	-3.6756	0.653	106	-5.633	<.0001
Th1 vs Th2	Fe	s_µ_g	1	-1.6994	0.653	106	-2.605	0.0105

Significant differences were observed primarily under simulated microgravity conditions. For no IR conditions, under simulated microgravity (s µ_g) and no HC (HC = 0 µM), Th1 cells exhibited significantly lower fold changes compared to Th2 cells (estimate = -1.9773, p < 0.0001), indicating nearly a two-fold reduction in gene expression. Similar trends were observed with 1 µM HC, where Th1 cells showed significantly lower fold changes than Th2 cells (estimate = -2.2003, p < 0.0001), again suggesting more than a two-fold decrease in gene expression. For the conditions where photon IR is present with simulated microgravity without HC, Th1 cells showed significantly lower fold changes compared to Th2 cells (estimate = -2.4927, p = 0.0002). This pattern persisted with HC, where Th1 cells had significantly lower fold changes than Th2 cells (estimate = -3.6756, p < 0.0001), indicating a more pronounced reduction. Under Fe irradiation and simulated microgravity with 1 µM HC, Th1 cells exhibited significantly lower fold changes compared to Th2 cells (estimate = -1.6994, p = 0.0105). However the estimate indicated a smaller effect on Th1 gene expression compared to the other contrasts. These findings suggest that under simulated microgravity conditions, Th1 cells consistently exhibit lower gene expression changes compared to Th2 cells, in line with what is observed for the cytokine levels measured in the previous section.

### 3.5 Transcriptome analysis highlights the unique expressed genes due to Fe ions exposure, HC, and simulated microgravity exposures

To better understand what the molecular mechanisms could be involved in these responses, RNA sequencing and proteomic investigations were employed. The following sections highlight the main results from this analysis.

Sequencing of the RNA obtained from CD4+ T cells of 4 donors was performed, for selected conditions: IR, HC, and simulated microgravity alone, as well as from cells exposed to the 3 simulated space stressors.

#### 3.5.1 Differential expression

**Figure 8:**
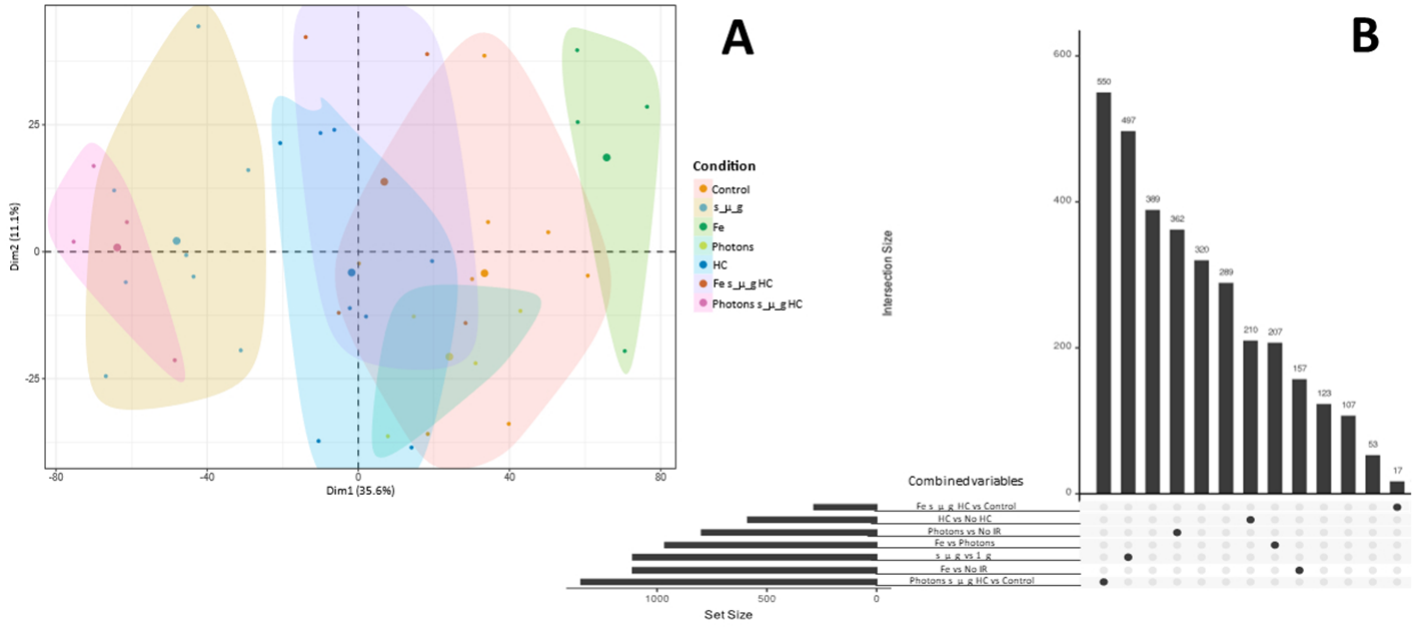
PCA and differential gene expression analysis upset plot. **(A)** Factorial map of the PCA separating bulk RNA sequencing gene expression data for each donor (each small dot). Large dot represents the mean for the samples in each group. The proportion of variance explained by each PCA is in parentheses. **(B)** Upset plot depicting the number of uniquely expressed genes for each condition comparing to control (No IR, 1_g, 0 µM HC) and the number of differentially expressed genes comparing Fe ion irradiation and photons irradiation. Data analyzed with Nextflow pipeline nf-core/differential abundance version 1.4.0. Data obtained from 4 donors.

**Figure 9:**
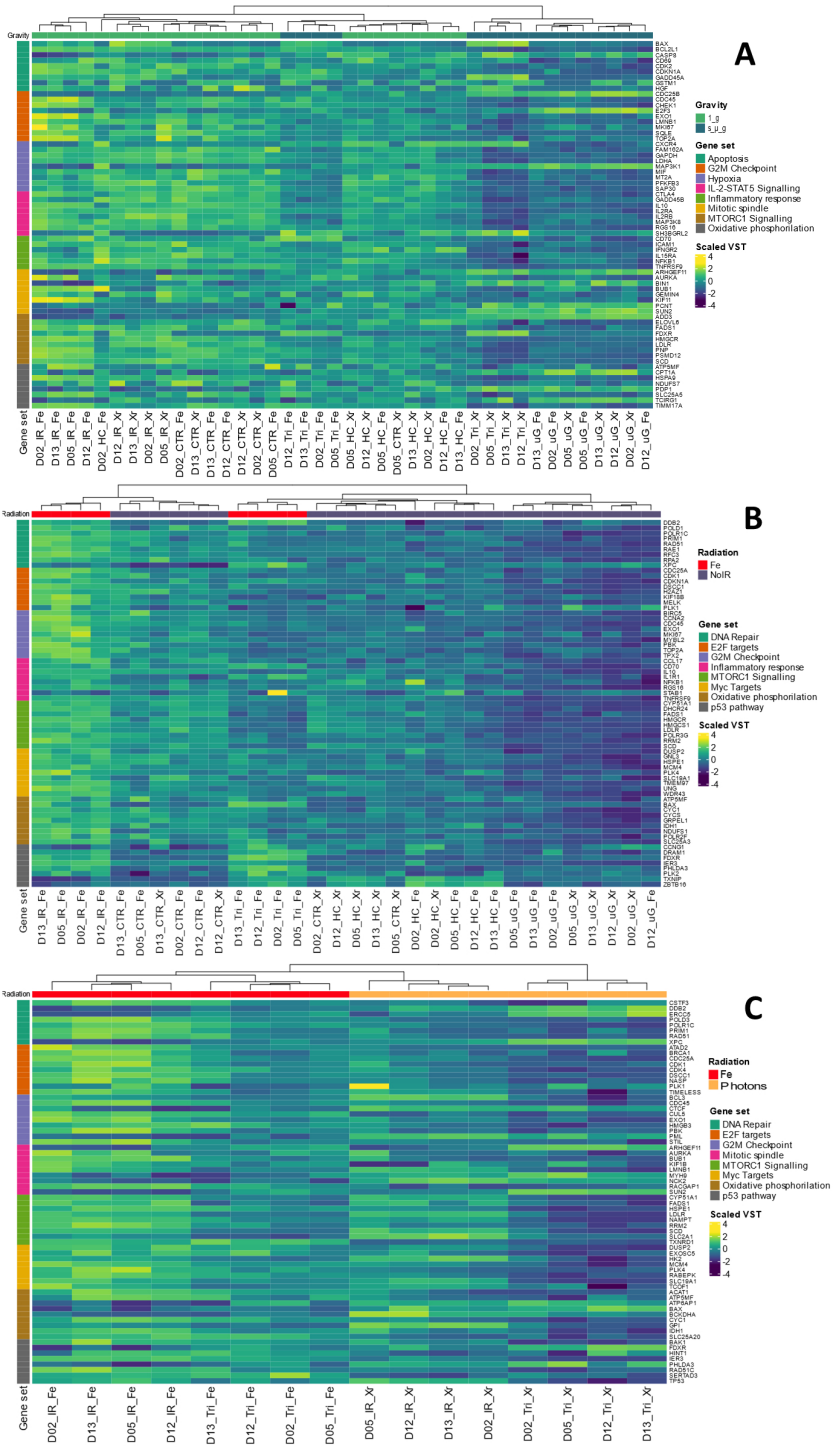
Heat maps for GSEA. **(A)** Contrast Simulated microgravity (s_µ_g) vs 1 g conditions. **(B)** Contrast Fe ions irradiation vs no irradiation (No IR) conditions. **(C)** Contrast Fe ions ve Photons. Dendrograms represent the sample clustering. Sample names represent donor number (D) and the condition (Fe, Iron ions; Xr, Photons; IR, Ionizing radiation; HC, Hydrocortisone; Tri, Triple exposure conditions (IR, HC and s_µ_g). Hallmarks collections are color coded as gene set. VST: Variance Stabilizing Transformation. Gene names are annotated on the right. Heatmaps created with Rstudio version 4.3.1. Data obtained from 4 donors.

Principal components analysis (PCA) of the RNA expression data highlights the inter-donor variability. However, clustering effects were observed for each of the groups (conditions) (**Figure 8A**). In addition, it can also be observed that the cluster for simulated microgravity and the combined triple exposure to photon irradiation, HC and simulated microgravity showed the largest deviation in RNA expression compared to the control cluster. This is further highlighted by the upset plot showcasing the largest differentially expressed genes for these two groups (497 and 550, respectively) as seen in **Figure 9B**. It is also important to highlight that Fe ions exposure induced the differential expression of 207 genes, compared to photon irradiation.

#### 3.5.2 Gene set enrichment analysis

GSEA was conducted to investigate the underlying pathways potentially involved in the alterations observed following exposure to single simulated space stressors. Specifically, the analysis focused on the role of simulated microgravity and Fe ion exposures. Heatmaps were generated for panel of genes selected from important hallmark gene sets that were found to be enriched for each of the contrasts studied. Genes were selected based on their ranking and role in each hallmark set.

**Figure 9A** depicts the heatmap contrasting the samples under the condition of simulated microgravity versus 1 g (including all conditions with simulated microgravity compared to all conditions without it). The results display Variance Stabilizing Transformation (VST) values for genes across eight different gene sets. Clustering patterns are evident among samples belonging to the same experimental condition groups: IR Fe, Photons IR (IR Xr), Control (CTR), HC, Tri (three simulated space conditions with either Fe IR or photons IR), and simulated microgravity (uG). This clustering can be seen at the bottom of **Figure 9A**, where each sample name represents the donor and the condition. Overall, the gene sets analyzed indicate a general downregulation of the majority of genes in the presence of simulated microgravity. Notably, the hallmark gene set for inflammatory response includes a collection of genes involved in various pathways related to inflammation regulation, such as genes encoding cytokines, growth factors and cell differentiation markers (40). NFKB1 is a subunit of the NF-kappa B (NF-κB) transcription factor complex, which plays a central role in regulating immune and inflammatory responses, cell survival, and proliferation. Downregulation of the NFKB1 gene is observed for the conditions with simulated microgravity, compared to the conditions without this simulates space condition, particularly under the additional exposure to photons and HC (Tri_Xr). The same can be said for intracellular adhesion molecule 1 (ICAM1) and interferon gamma receptor subunit 2 (IFNGR2). Additionally, the genes in the mTORC1 signaling hallmark, which is crucial for T cell activation, showed a significant downregulation in simulated microgravity conditions (41). For the hallmark gene sets regarding apoptosis it is interesting to observe that the caspase 8 (CASP8) gene displays upregulation under the conditions with simulated microgravity compared to lower levels of VST for the conditions without this factor, particularly when simulated microgravity was the only space stressor used. Other genes displaying this behavior are E2F Transcription Factor 3 (E2F3) gene from the E2F Targets hallmark; the Rho Guanine Nucleotide Exchange Factor 11 (ARHGEF11), Bridging Integrator 1 (BIN1), Pericentrin (PCNT), and Sad1 And UNC84 Domain Containing 2 (SUN2) from the mitotic spindle hallmark; Mitogen-Activated Protein Kinase Kinase Kinase 8 (MAP3K8) gene from the hypoxia hallmark; and Carnitine Palmitoyltransferase 1A (CPT1A), Pyruvate Dehydrogenase Phosphatase Catalytic Subunit 1 (PDP1), and T Cell Immune Regulator 1 (TCIRG1) from the oxidative phosphorylation hallmark.

Fe ions are a significant component of galactic cosmic rays (GCR), contributing to the biological effects of exposure to ionizing radiation (IR) during space exploration. We observed in previous sections that there is an upregulation of Th1 cytokines in the presence of Fe ions irradiation. **Figure 9B** shows the heatmap obtained for several enriched hallmark gene sets, contrasting conditions with Fe ions irradiation versus those without IR. Similar to previous results, the clustering of samples by experimental group is evident in this contrast. Genes related to the DNA Repair hallmark gene set are upregulated in the Fe ions irradiation condition. However, this upregulation is less pronounced when Fe ion irradiation is combined with simulated microgravity and HC exposure. Under simulated microgravity conditions, the lowest values of VST are observed for the majority of genes. The Xeroderma Pigmentosum, Complementation Group C (XPC) gene, involved in DNA damage sensing and nucleotide excision repair (NER), is downregulated under Fe ions irradiation alone but upregulated when simulated microgravity is present. This suggests a complex interaction between IR and microgravity on DNA repair mechanisms. Similarly, hallmark gene sets for E2F Targets, Inflammatory Response, mTORC1 Signaling Pathway, and Oxidative Phosphorylation were enriched in this dataset. The selected genes also show upregulation in conditions with Fe ions IR, which is attenuated by the presence of other simulated space conditions, especially simulated microgravity. MYC-regulated genes, which are involved in various cellular processes including cell cycle progression, metabolism, and apoptosis, were also upregulated. This upregulation may promote cell survival and proliferation in response to radiation-induced damage. Again, Fe ions IR induces upregulation of genes in this hallmark set, while the presence of other simulated space stressors, like HC and simulated microgravity, reduces this upregulation. The heatmap also highlights the upregulation of genes in the p53 pathway hallmark under conditions involving HC exposure. Genes such as Thioredoxin Interacting Protein (TXNIP) and BTB Domain and CNC Homolog 1 (BCL6B) show increased expression, indicating activation of p53-mediated pathways in response to combined stressors.

**Figure 9C** shows the heatmap generated when contrasting the two types of IR utilized in this study. High Linear Energy Transfer (LET) IR, such as Fe ions, interacts with biological matter in different manner than low LET IR, such as photons (42). GSEA revealed the enrichment of several hallmark gene sets related to DNA damage and repair (DNA Repair, E2F Targets, p53 Pathway), cell cycle management (G2M Checkpoints), cell division (Mitotic Spindle), cell proliferation (mTORC1, MYC Targets), and metabolism pathways (Oxidative Phosphorylation, MYC Targets). The dendrograms show clear clustering of the experimental groups. Generally, the upregulation of genes within each hallmark gene set is more pronounced under Fe ion irradiation conditions. However, the combination of Fe ion irradiation with other simulated space conditions (samples with Tri_Fe) shows an overall decrease in gene upregulation, suggesting that additional factors influence gene expression. Similarly, conditions where photon irradiation is combined with other simulated space stressors display a pronounced downregulation of genes across all hallmark gene sets. Interestingly, some genes in the DNA Repair pathway, such as Damage-Specific DNA Binding Protein 2 (DDB2), Excision Repair Cross-Complementation Group 5 (ERCC5), and again Xeroderma Pigmentosum, Complementation Group C (XPC), are more upregulated in the Tri conditions compared to conditions with radiation alone (either Fe ions or photons). This suggests the activation of different DNA repair mechanisms depending on the radiation type and the presence of other simulated space conditions. In the G2M Checkpoint hallmark, genes such as B-Cell Lymphoma 3 (BCL3), Cyclin C (CCNC), and Promyelocytic Leukemia (PML) are downregulated under Fe ion irradiation conditions compared to photon irradiation conditions. Similarly, Myosin Heavy Chain 9 (MYH9) and NCK Adaptor Protein 2 (NCK2) genes in the Mitotic Spindle hallmark gene set also show downregulation under Fe ion irradiation compared to photon irradiation. Oxidative phosphorylation hallmark genes also display a contrast between Fe ion irradiation and photon irradiation. Genes such as ATPase H+ Transporting Accessory Protein 1 (ATP6AP1), BCL2 Associated X, Apoptosis Regulator (BAX), and Branched Chain Ketoacid Dehydrogenase E1 Subunit Alpha (BCKDHA) were downregulated in Fe ion conditions compared to photon irradiation.

While gene expression analysis provides critical insights into the transcriptional changes induced by various space stressors, it is also essential to understand how these changes are translated into the proteome—the complete set of proteins expressed by a cell, tissue, or organism. Proteins are the functional molecules that carry out the majority of cellular processes, and their expression levels, modifications, and interactions ultimately determine the physiological response to environmental conditions.

## 4 Discussion

Space is a hostile environment that induces numerous changes in the health of astronauts. With the rise in efforts to increase human presence in space, looking towards a permanent presence on the Moon, and advancing further to Mars, a better understanding of the mechanisms behind health changes and the development of appropriate countermeasures is paramount. The development of space-related immune dysfunction has been documented since the apollo era, with reports of suppressed immune function. Therefore, understanding the nature of this altered immunological state is extremely important to allow safer space missions. CD4+ T cells are important orchestrators of the immune response; however, the effects of the space environment on their behavior are yet to be elucidated.

Access to either astronauts’ samples or real-space conditions for performing experiments is limited. The use of simulation platforms can provide valuable insights into the effects of simulated microgravity, elevated stress hormone levels, and radiation on CD4+ T-cells. This allows researchers to mimic the physical conditions of space and study how they affect T cell function, cytokine production, and immune response. In the context of spaceflight, astronauts are exposed to all three main space stressors, including microgravity, radiation, and psychological stress, at the same time. It is still unknown what contribution to the observed immune dysfunction is given due to each individual stressor or the combined effects of all three stressors. Previous research has been conducted on Jurkat cells, an immortalized leukemic cell line, including different combinations of simulated space conditions, and highlighted the impact of simulated microgravity and HC on IL-2 levels. The results showed that exposure to simulated microgravity and HC strongly suppressed IL-2 secretion from the stimulated cells (43). Taking the same concept, with the goal of elucidating the specific mechanisms by which T cells are affected by these stressors, the present study aimed to use donor-derived CD4+ T cells, employing a multimodal approach ranging from phenotypic analysis to mechanistic investigations.

### 4.1 Cytokine profiles after exposure to simulated space conditions in different combinations

To our knowledge, this work is the first to explore the effects of different combinations of simulated space conditions on CD4+ T cells extracted from healthy male volunteers. The findings of this study indicate that simulated space conditions, including microgravity, exposure to IR, and elevated stress levels, have a significant impact on the production of cytokines by CD4+ T cells and the balance between Th profiles, particularly Th1 and Th2 subtypes. These results highlight the complex interactions between these factors and suggest that they play a crucial role in modulating cytokine levels.

Our results showed that an acute exposure to 1 Gy IR increased the levels of all cytokines measured, 24h after exposure. This generalized increase in all evaluated cytokines can be an indication of the presence of cellular mechanisms to manage the response to IR exposure. Pro-inflammatory cytokines (IFN-γ, TNF-α, and IL-2) occur at an early stage in response to IR. These cytokines are normally associated with the production of reactive oxygen species (ROS) and reactive nitrogen species (RNS). As a result, an additive effect on ROS and RNS produced by IR perpetuates the cell’s DNA damage response (DDR). Anti-inflammatory cytokines (such as IL-4, IL-5, and IL-13) are triggered to act in order to re-establish homeostasis. The equilibrium between pro- and anti-inflammatory responses occurs in a wave-like manner. The observed results at 24h after radiation exposure might depict a unique moment in time, that captures cumulative results of both the initial pro-inflammatory response and the consequent anti-inflammatory reaction to the former. Studies have revealed that irradiation triggers a complex response in cytokine production, involving both pro-inflammatory and anti-inflammatory cytokines. The specific pattern of this response is highly dependent on factors such as the study model, irradiation characteristics (type, dose, and dose rate), time after exposure, and size and location of the irradiated area (44).

Definitions of a low dose vary, and the specific threshold depends on the context. The International Committee for Radiation Protection (ICRP) suggests that a dose lower than 100 millisieverts (mSv) poses a very low carcinogenic risk (45). Studies on mice have shown that low doses of IR led to a Th1 type immune response, whereas high doses let to a Th2-type response (46). Epidemiological studies have depicted a Th2 phenotype in populations living in areas with elevated background radiation (typically low dose rate) (47). A 2Gy total body irradiation in mice primed the CD4+ T cell population to a higher tumor infiltration, characteristic of Th1 phenotypes (48). Additional to the dose, the type of ionizing radiation is also a factor to consider in terms of the biological response. Low LET radiation, such as photons, deposits the energy in a sparse manner whereas for Fe ion irradiation (high LET) ionization tracks are close together, which could lead to clusters of damage which are difficult to repair (42). This is an important factor contributing to the elevated relative radiobiological effectiveness (RBE) of HZE particles. Research efforts on T cells are ongoing to better understand the specific effects of exposure to different doses and LET types of radiation, with the majority of the data coming from epidemiological studies (49). Although evidence shows that low-to-moderate doses of both low and high LET prime an anti-inflammatory phenotype, long-term studies have shown that exposure to very low doses of IR (either acute or chronic) has a pro-inflammatory effect (50). The Luminex results presented in this paper indicate a stronger cytokine release after an acute 1 Gy Fe ions exposure, compared to an acute 1 Gy photon exposure. This was validated in the linear regression models, where photon irradiation was not found to be a significant predictor of the response. On the other hand, Fe ions was a high statistically significant predictor of the response. Fe ions are an important component of the GCR radiobiological impact due to the complex damage caused by the interaction of the Fe particles with the biological matter. High LET IR has been shown to trigger an intricate DDR that in the context of the immune system, creates a pro-inflammatory background that will further activate the DDR. High LET radiation can trigger a stronger initial pro inflammatory response. However, there are many uncertainties regarding the precise mechanisms and outcomes of high LET radiation exposure on the immune system.

The suppressive effects of microgravity exposure on the activation of T cells are fairly documented (22). Previous work conducted on Jurkat cells has demonstrated the impact on IL-2 production after stimulation on cells exposed to 24h simulated microgravity, showing a decrease of more than 50% of the normalized cytokine level compared o the control conditions (43). In this study several cytokines for Th subtypes were evaluated, allowing for a more robust assessment of the effects on the T cell phenotypes. The grouped analysis of the cytokines for the Th1 and Th2 subtypes, showed that all the groups where simulated microgravity was present displayed an apparent favoring of Th2 phenotype in detriment of the other Th subtypes. While IFN-γ acts as a suppressor of Th2 activity, low levels of this cytokine might explain why simulated microgravity conditions, that diminished the Th1 cytokines, might show a Th2 favoring (51). This has been previously demonstrated during shuttle and International Space Station (ISS) missions, where activated peripheral blood T cells showed a lower IFN-γ to IL-10 ratio, suggesting a Th2 shift (52).

In this paper, we have shown that HC exposure alone leads to the suppression of all cytokines measured. Glucocorticoids, like cortisol, are known immune regulators that can induce Th1 cell suppression while promoting Th2 activity, reducing pro-inflammatory states (53). Cortisol is also known to exert a nonlinear dose-response on the cells of the innate immune system. Rather, a ‘bi-phasic’ regulation of inflammation is proposed, with lower doses contributing to an activation of inflammatory responses and higher does to a suppression of the inflammatory response (54). It might be that the overall effect of HC exposure relates to the dose used in this experimental setup (1 µM), impacting all cytokines independent of the phenotype. Chronic stress has also been suggested to have implications on the outcome of radiotherapy treatments through the suppression of the immune system (55, 56). Interestingly, exposure to simulated microgravity alone reduced cytokine levels to a higher degree than exposure to HC alone. This might indicate a difference in the mechanisms of T cell suppression in these two components of the space environment.

### 4.2 Interaction effects of the simulated space stressors, predicting the effect with linear regression models

In space, exposure to the entire spectrum of stressors is present, making it difficult to pinpoint which environmental factors cause these alterations. The use of ground-based platforms permits the design of intricate experimental setups in which different combinations of each simulated space stressor can be achieved. The experimental setup used in this study allowed for the exposure of CD4+ T cells to different combinations of simulated space stressors. This combined analysis showed that exposure to IR, particularly Fe ions, counteracted the suppressive effects of exposure to either simulated microgravity or HC. Alternatively, combining IR (both photons and Fe ions) with either HC or microgravity, the cytokine-stimulatory effect of radiation exposure was suppressed. Another way of observing this picture is by saying that an acute 1 Gy of IR exposure tends to counteract the suppressive effects of HC or microgravity exposure. The linear regression analysis of these complex interaction effects seems to corroborate this finding. The groups where both HC and microgravity were present showed that the addition of simulated microgravity to the setup further decreased the levels of cytokines to levels similar to those measured in the groups with only simulated microgravity exposure. Of note, the interaction of the factors was not significant for all the cytokines (in contrast to the high significance of the single exposure conditions). This might represent the different mechanisms taking place when the simulated space conditions are present. A review by Moreno-Villanueva et al. has also highlighted the possible interaction effects between IR and microgravity and its impact on DDR, hinting the need for more studies that tackle this interactions. Radstake et al. has previously demonstrated the complex interactions between different combinations of simulated space conditions, including HC, in a fibroblast *in vitro* model (57).

### 4.3 Mechanistic insights with transcriptomics

The observed complex interaction effects can be better elucidated with mechanistic assays such as transcriptomics. The results from our transcriptomics data showed that the exposure to the different simulated space conditions, induced the differential expression of unique genes (not shared by other conditions),indicating the potential activation of unique pathways. The GSEA performed yielded a group of hallmark gene sets that were found enriched for the different contrasts analyzed. There is a notable trend of a general downregulation in the majority of genes under simulated microgravity conditions. This indicates a potential suppression of various cellular activities and processes in response to this specific stressor. The hallmark gene set for inflammatory response, including key genes such as NFKB1, ICAM1, and IFNGR2, shows a significant downregulation. This suggests that simulated microgravity may dampen the inflammatory response, potentially affecting immune regulation and inflammation-related pathways and favoring Th2 differentiation, since Th1 and Th2 pathways are mutually inhibitory. The mechanisms by which microgravity suppresses T cell activity are subject of scrutiny, with a definite model still being formulated (58–60). The downregulation of genes in the mTORC1 signaling pathway highlights an impact on T cell activation and function, possibly also promoting a Th2 response, since mTORC1 signaling is involved in Th1 differentiation through metabolic programming (61). RT-qPCR showed also a shift toward Th2 profiles via the GATA3 transcription factor, adding another layer to the mechanisms behind this shift in response to simulated microgravity exposure. Interestingly, genes involved in apoptosis, such as CASP8, show upregulation under simulated microgravity. This upregulation suggests a possible increase in apoptotic activity, which could be a cellular response to stress. Of note that flow cytometry data on cell viability showed no differences in viable cell percentage compared to control conditions. Previous studies have demonstrated that simulated microgravity might play a role in apoptotic pathways in cancer cells, related to mitochondria and ROS (62). It might be that these altered gene expression profiles are related to mitochondrial activity and cellular energy status. Genes from the oxidative phosphorylation hallmark, including CPT1A, PDP1, and TCIRG1, also exhibit notable expression changes. These alterations suggest that simulated microgravity may affect cellular energy metabolism and mitochondrial function. Additionally, the upregulation of genes from the E2F Targets hallmark, and the mitotic spindle hallmark indicates alterations in cell cycle regulation and mitosis. Previous research has also showed the on the effect on the mitotic spindle assembly (63). Taken together, it is clear that a myriad of pathways ae involved in the response of the CD4+ T cells to simulated microgravity contributing to the overall suppression of T cell activity.

Our transcriptomic analysis identified the upregulation of genes involved in DNA repair, such as DDB2 and ERCC5, in response to Fe ion irradiation. This suggests an enhanced capacity for DNA damage recognition and repair, which is crucial for maintaining genomic integrity under high-energy particle exposure (64). Interestingly, XPC was downregulated under Fe ions alone but upregulated in the presence of simulated microgravity, indicating a complex interplay between different space stressors in modulating DNA repair pathways. Studies conducted in *C. Elegans* indicated an upregulation of genes in the NER, base excision repair (BER) and mismatch repair (MMR), in response to microgravity (65). On the contrary, a study on lymphocytes exposed to simulated microgravity in rotating wall vessels, displayed downregulation DNA repair genes, particularly, of the MMR pathway (66).

The comparative analysis of high LET IR (Fe ions) and low LET IR (photons) yields several important conclusions regarding the biological effects of these different types of radiation in the context of simulated space conditions. High LET IR, such as Fe ions, generally causes a more pronounced upregulation of genes involved in DNA damage and repair, cell cycle management, cell division, cell proliferation, and metabolic pathways compared to low LET IR (photons). This indicates a stronger biological response to high LET radiation. The presence of additional simulated space conditions (e.g., microgravity and HC exposure) significantly modulates the gene expression response to both types of IR. Specifically, the combination of Fe ion irradiation with other space stressors (Tri_Fe) results in a decrease in the upregulation of genes across multiple hallmark gene sets. Due to the fact that the HC impact on the gene expression profiles is not as strong may indicate that the main factor contributing for this downregulation is simulated microgravity.

### 4.4 Limitations and future perspectives

Simulated microgravity platforms such as the RPM may introduce shear stress onto the experimental design. The inhouse developed model that creates an airtight cell culture system resulted in a lack of oxygenation and CO2 buffering in the cell culture media. To address concerns about cell toxicity, we limited our exposure time to 24 hours. The use of a higher dose of hydrocortisone was necessary for this short time of exposure, which has been shown to have observable effects (43). Some studies have found that astronauts’ plasma cortisol levels can reach as high as 0.610 µmol/L during space flight (67). His concentration of cortisol surpasses the threshold suggested by the inverted U model, where levels above ~0.45 µgl/dL (~1µmol/L) can be detrimental (55, 68). Moving forward, it may be beneficial to explore different hydrocortisone concentrations to establish a threshold for the observed alterations at 1 µM. This could be particularly useful for monitoring astronauts’ cortisol levels during long-duration space missions.

In the future, missions to Mars will expose astronauts to approximately 1 Sv of IR radiation, which is equivalent to 1 Gy of X-ray exposure and 0.4 Gy of GCR exposure (69). The isoeffective dose for higher LET irradiations of immune cells is currently unknown, so it was chosen to use the same deposition of energy (1 Gy) for both types of irradiation. The significance of the Fe ion component in GCRs regarding radiobiological effectiveness is well-known. By comparing it to photon irradiation, this setup allowed us to explore the LET-specific differences in the response of CD4+ T cells. Future investigations should aim to use more space-relevant ions or protons, or ideally, GCR simulators such as those available at the NASA space radiation laboratory (70) or at GSI Helmholtzzentrum für Schwerionenforschung (71). In this study, IR exposure was achieved using two different experimental setups due to the nature of the radiation beam lines. While the total dose applied was 1 Gy at a dose rate of approximately 0.5 Gy/min, the deposition of Fe ions was less constant than that of photon irradiation, affecting the dose rate and the distribution of the particles through the samples, in comparison with the uniform field achieved during photon irradiation. This might have implications also for the effects observed. As mentioned, in space the accumulated dose (on a Mars round trip of 500 for example) would be approximately 1 Sv, with relatively low dose rates (1.84 mSv/day) (72). Another limitation in this 24 h setup, is that it calls for an acute irradiation to be administered. Future works should consider lower dose rates that better match dose rates found in space and can be achieved in ground-based facilities, using, for example, *in vivo* models (73).

Some assays conducted for this study have also showed some shortcomings. The analysis of cytokines in brefeldin A treated cells through flow cytometry proved inconclusive. This method of staining cytokines that are retained in the cells might not be the most adequate. Nevertheless, flow cytometry is an important tool for studying T cell phenotypes. Future work should focus on staining intracellular transcription factors that are characteristic of T cell lineages, avoiding the disruption of protein trafficking that could impair the results. Proteomic investigation was hindered by the low protein yield obtained from the samples, leading to very little information on protein fold changes. Future work should adapt the protein extraction and purification protocols to address this issue. This study also focused the analysis on cytokines from the Th1 and Th2 subtypes of CD4+ t cells. Treg and Th17 is another important subtype that plays a crucial role in immunity (11). Future experiments should include this analysis, providing a wider scope on the effects of simulated space conditions on T cell fates.

In this study, cell numbers were quantified for apoptosis assays to ensure uniformity in the analysis, with flow cytometry used to count the same number of events for each condition. However, for other assays, although initial cell counts were identical at the onset of each experiment, we did not perform subsequent cell counts post-exposure. This approach was taken based on our experimental design, which prioritized the assessment of functional and biochemical changes over cell viability or proliferation post-treatment, except in the context of apoptosis. We acknowledge that this could impact the interpretation of our results, as changes in cell viability and proliferation can influence biochemical and functional assay outcomes. Recognizing the importance of this data, this is seen as a limitation of our current study. Future experiments plan to include comprehensive cell counting post-exposure for all relevant assays to better correlate functional changes with alterations in cell numbers. This will enhance the robustness of the data and provide a more comprehensive understanding of the impact of simulated space conditions on cellular systems.

Isolated immune cells were used to perform this study. The immune system is comprised of a complex network of different cellular components that work together and play inter-regulatory roles. It is therefore important to note that insights from this study have clear limitations in terms of extrapolating for general immune effects. For example, cytokine levels have been shown to vary depending on the experimental model, with factors such as media serum or plastic affecting the cytokines expressed by macrophages (74). Future work could focus on the use of models that better represent the complex environmental context that immune cells are exposed to. The use of 3D cell culture models such as organs-on-chips could prove useful to study the role of immune cells in the pathophysiology of several space-related dysfunctions. Murine models with simulated microgravity (hind limb unloading (HLU)) in combination with IR and HC exposures could also be of important use for capturing the complexity and interconnectedness of the immune system response to spaceflight conditions. Additionally studies involving immune cells collected from volunteers in head-tilt bed rest or dry immersion studies could also help elucidate, from a holistic perspective, the gravity unloading effects on human health.

Additionally, the separation of Th cells was performed before stimulation with soluble anti-CD3/CD28 antibodies to mimic immunogenic challenges. While this approach allows for controlled and reproducible stimulation of T cells, it does not fully replicate the complexity of CD4+ T cell activation *in vivo*. T cell activation in the natural environment involves multiple co-receptors and co-stimulatory signals provided by antigen-presenting cells (APCs). These co-stimulatory signals, along with cytokines secreted by APCs, are critical for the proper polarization and differentiation of T helper (Th) cell subsets (75). The use of soluble antibodies, as opposed to surface-bound antibodies, does not form an immune synapse, which is essential for robust T cell activation and signaling (76). Consequently, the absence of these co-stimulatory signals in this model may have impacted the polarization and functional responses of Th cells. Moreover, the separation of Th cells before stimulation removes them from their natural context, potentially altering their behavior and responses. Future studies will aim to incorporate more physiologically relevant models of T cell activation, such as using APCs to provide a more comprehensive set of co-stimulatory signals and cytokines. This approach would better replicate the *in vivo* environment and potentially yield more accurate insights into the behavior of Th cells under simulated space flight conditions.

A group of 21 male volunteers was used for the isolation of CD4 + T cells. Results showed that some degree of interdonor variability exists. This highlights the importance of personalized medicine for the future of risk assessment and countermeasure development (77). An important factor to consider is the biological sex differences to the space environment. The immune system is no exception to the gap in knowledge as there is a clear need to address sex differences in immunological studies (78). It is already established that spaceflight might affect women differently, with higher risks, namely in respect to the reproductive system effects, hormonal changes in flight and genitourinary (79). Including females in study designs is now imperative for several national agencies, such as the European Space Agency SA VIVALDI I dry immersion study (80). Using the experimental setup described in this study, future work should perform these analysis on a pool of female volunteers, and address any sex specific differences in the response of CD4+ T cells that might arise.

The inclusion of a broad age spectrum (from 23 to 53 years) could introduce significant variability in immune function, potentially influencing the outcomes of this study. Age-related changes in the immune system, known as immunosenescence, are characterized by a decline in the function and number of T cells, altered cytokine profiles, and decreased response to vaccinations and pathogens (81). These changes could differentially impact the immune responses to the simulated spaceflight conditions in our study, particularly among older participants. Moreover, research suggests that andropause, which may begin as early as the fifth decade of life, could also affect immune function through hormonal changes, primarily due to decreasing testosterone levels (82). Testosterone has been shown to influence several components of the immune system, including cytokine production and T cell responses (83). Considering these factors, future studies should aim to narrow the age range or consider stratifying results by age to more precisely evaluate the impacts of spaceflight-like conditions on immune function. This stratification could enhance our understanding of age-specific immune changes and improve the relevance of our findings to active astronauts, who are typically within a narrower age range. Plans to further investigated these aspects in subsequent studies will refine our understanding of age-related immune modulation in spaceflight contexts.

During volunteer selection, participants were screened for a comprehensive list of immune-related disorders and infectious diseases to minimize confounding immune responses. This approach aimed to ensure that the measured immune functions accurately reflected the effects of the simulated space flight conditions. However, it is important not explicitly controlling for alcohol consumption, allergies, and antihistamine use represents a limitation. These factors can significantly influence immune function, and their exclusion in future studies will enhance the robustness of our findings. Specifically, chronic alcohol consumption can suppress immune function (84), while antihistamines can modulate immune responses by affecting cytokine production and immune cell activity (85). Addressing these variables will provide a clearer understanding of immune function in simulated spaceflight conditions. While allergies were not explicitly included as exclusion criteria, this factor will be considered in future studies to avoid potential confounding effects.

Data normalization to the control condition for each donor was employed to mitigate the influence of interdonor variability. This approach allows for a consistent baseline across different experimental conditions, facilitating more accurate comparisons of the relative changes induced by the treatments. While presenting data as relative numbers can be subject to artificial exaggeration, normalizing to the control condition for each donor helps to reduce this risk by focusing on changes that are directly attributable to the experimental interventions. Normalizing data in this manner is a common practice in immunological research, as it accounts for the inherent variability among different individuals. This method enhances the reliability of detecting true experimental effects, providing a clearer understanding of how the experimental conditions impact immune responses. Future studies may further benefit from additional normalization techniques, such as log or square root transformations, to complement this approach and provide a more comprehensive analysis of the data.

Finally, in conducting this study, sample availability was challenging and necessitated the use of a limited number of donor cells in certain assays. While this approach allowed for the exploration of immune responses across a diverse set of conditions, it also introduces limitations regarding the number of observations that could significantly impact the statistical power and variability of our results. Recognizing these constraints, the need for caution in interpreting these findings should be emphasized and future studies will aim to include a broader range of samples to enhance the robustness and reproducibility of the results. Such studies would be invaluable in confirming the trends observed in the present study and in further elucidating the complex dynamics of immune responses under simulated space conditions.

To the best of our knowledge, this study is the first to expose healthy donor-derived CD4+ T cells to different combinations of simulated space conditions and employ a comprehensive multimodal approach to study phenotypic and mechanistic changes. The main goal was to provide insights into the role of CD4 + T cells in the context of spaceflight conditions and how they can be connected to documented alterations of the immune system. The findings of this research may have implications for the development of countermeasures to protect the health of astronauts during long-duration spaceflights. For example, the identification of key genes and pathways involved in T cell activation and immune responses could potentially lead to the development of immune boosters or targeted therapies to mitigate the negative effects of space stressors on immune function. Additionally, understanding how the individual stressors of microgravity, radiation, and psychological stress interact with each other to impact T cell functionality can guide the development of holistic countermeasures that address multiple stressors simultaneously. Moreover, directing research efforts towards the immune system in response to different environmental factors, such as HC and IR, might also provide a basis for a better understanding of T cell responses in disease, stress, or radiotherapy contexts.

## Data Availability

The datasets presented in this study can be found in online repositories. The names of the repository/repositories and accession number(s) can be found below: E-MTAB-14151 (Array Express) https://www.ebi.ac.uk/biostudies/arrayexpress/studies/E-MTAB-14151?key=45d964db-d2ff-429e-90d9-969e02aba3c3.
