# Supplementary material for: Effects of simulated space conditions on CD4+ T cells: a multi modal analysis
Source: Front Immunol. 2024 Sep 2;15:1443936. doi: 10.3389/fimmu.2024.1443936 (PMC11402665; doi:10.3389/fimmu.2024.1443936)
Supplement: Supplementary file 1 [file DataSheet1.docx]

Supplementary Material

Effects Of Simulated Space Conditions On CD4+ T Cells: A Multi Modal Analysis

**Silvana Miranda^2^, Randy Vermeesen, Ann Jansen, Emil Renhberg, Emre Etlioglu, Sarah Baatout, Kevin Tabury, and Bjorn Baselet***

***Correspondence:** Corresponding Author: bbaselet@sckcen.be

# Supplementary Materials

## Volunteer inclusion/Exclusion criteria

### Inclusion criteria

The criteria for the inclusion of subjects in this study are:

- Male individuals
- Age interval: 18 to 55 years
- No known history of immune or immune related disorders
- No immune‐modulatory medication in the 6 months prior to the participation in the study
- No infection (sneeze, cold,…) in the 2 weeks prior to the participation in the study
- Non‐smokers
- Signed informed consent
- Medium to high CD4+ T cell count after isolation procedure: ≥0.3 million CD4+ T cells per 1ml
- of blood

### Exclusion criteria

Exclusion criteria include

- Female individuals
- Ages interval: under 18 and above 55 years
- Documented or known history of immune or immune related disorders
- The use of immune‐modulatory medication in the past 6 months
- Infection (sneeze, cold,…) in the 2 weeks prior to the participation in the study
- Smokers
- Not having a signed informed consent
- Low CD4+ T cell count after isolation procedure: <0.3 million CD4+ T cells per 1ml of blood

## Population analysis

### Age Distribution – R analysis (RStudio verison4.3.1)

# Load data
Data_donors_pop <- read_excel("Master_file_results.xlsx", sheet = 1)
# Obtain information about the dataset
head(Data_donors_pop)

## # A tibble: 6 × 17
## `Donor code` Assay Luminex Age RBC HCT HGB WBC BASO EO LYMPH
## <chr> <chr> <chr> <dbl> <dbl> <dbl> <dbl> <dbl> <dbl> <dbl> <dbl>
## 1 FIND001 P Y 46 4.89 44.4 14.8 4.05 0.5 2 29.6
## 2 FIND002 T Y 35 5.96 51.8 17.2 7.97 0.4 3 25.3
## 3 FIND003 FC N 39 5.48 47.3 16.2 6.32 0.5 1.6 39.7
## 4 FIND004 FC N 32 5.75 49.2 17 7.13 0.3 1.5 32.3
## 5 FIND005 T Y 27 5.24 45.5 15.9 3.7 0.3 1.4 46.5
## 6 FIND010 P Y 24 4.95 44.7 15.6 5.11 0.2 1 27.8
## # ℹ 6 more variables: MONO <dbl>, NEUT <dbl>, MCH <dbl>, MCHC <dbl>, MCV <dbl>,
## # PLT <dbl>

summary(Data_donors_pop)

## Donor code Assay Luminex Age
## Length:21 Length:21 Length:21 Min. :23.00
## Class :character Class :character Class :character 1st Qu.:32.00
## Mode :character Mode :character Mode :character Median :36.00
## Mean :37.52
## 3rd Qu.:45.00
## Max. :53.00
##
## RBC HCT HGB WBC
## Min. :4.540 Min. :41.50 Min. :13.70 Min. :3.700
## 1st Qu.:4.930 1st Qu.:43.90 1st Qu.:14.80 1st Qu.:5.330
## Median :5.090 Median :44.70 Median :15.60 Median :5.540
## Mean :5.178 Mean :45.28 Mean :15.42 Mean :5.921
## 3rd Qu.:5.290 3rd Qu.:46.60 3rd Qu.:15.90 3rd Qu.:6.930
## Max. :5.960 Max. :51.80 Max. :17.20 Max. :7.970
##
## BASO EO LYMPH MONO
## Min. :0.2000 Min. : 0.800 Min. :17.90 Min. : 6.50
## 1st Qu.:0.3000 1st Qu.: 1.500 1st Qu.:27.80 1st Qu.: 7.80
## Median :0.4000 Median : 2.700 Median :29.50 Median : 9.30
## Mean :0.4476 Mean : 3.062 Mean :30.45 Mean :11.84
## 3rd Qu.:0.5000 3rd Qu.: 3.100 3rd Qu.:35.60 3rd Qu.:12.40
## Max. :1.1000 Max. :12.100 Max. :46.50 Max. :53.10
##
## NEUT MCH MCHC MCV
## Min. :39.60 Min. :27.80 Min. :33.00 Min. :84.10
## 1st Qu.:49.50 1st Qu.:28.90 1st Qu.:33.40 1st Qu.:85.70
## Median :57.05 Median :29.70 Median :34.10 Median :86.80
## Mean :56.42 Mean :29.82 Mean :34.06 Mean :87.52
## 3rd Qu.:62.30 3rd Qu.:30.30 3rd Qu.:34.40 3rd Qu.:89.70
## Max. :74.20 Max. :32.20 Max. :35.70 Max. :93.80
## NA's :1
## PLT
## Min. :173.0
## 1st Qu.:206.0
## Median :232.0
## Mean :227.7
## 3rd Qu.:244.0
## Max. :284.0
##

str(Data_donors_pop)

## tibble [21 × 17] (S3: tbl_df/tbl/data.frame)
## $ Donor code: chr [1:21] "FIND001" "FIND002" "FIND003" "FIND004" ...
## $ Assay : chr [1:21] "P" "T" "FC" "FC" ...
## $ Luminex : chr [1:21] "Y" "Y" "N" "N" ...
## $ Age : num [1:21] 46 35 39 32 27 24 29 45 48 50 ...
## $ RBC : num [1:21] 4.89 5.96 5.48 5.75 5.24 4.95 5.29 4.54 5.11 4.91 ...
## $ HCT : num [1:21] 44.4 51.8 47.3 49.2 45.5 44.7 46.6 42.6 46.5 44.2 ...
## $ HGB : num [1:21] 14.8 17.2 16.2 17 15.9 15.6 15.9 14.6 15.6 15.2 ...
## $ WBC : num [1:21] 4.05 7.97 6.32 7.13 3.7 5.11 7.38 4.99 7.77 5.54 ...
## $ BASO : num [1:21] 0.5 0.4 0.5 0.3 0.3 0.2 0.4 0.4 0.3 0.5 ...
## $ EO : num [1:21] 2 3 1.6 1.5 1.4 1 1.6 8.4 0.9 2.7 ...
## $ LYMPH : num [1:21] 29.6 25.3 39.7 32.3 46.5 27.8 29.4 29.1 17.9 36.6 ...
## $ MONO : num [1:21] 7.4 9.3 12 8.3 12.2 7.8 7.2 9 8.9 8.3 ...
## $ NEUT : num [1:21] 60.5 62 46.2 57.6 39.6 63.2 61.4 NA 72 51.9 ...
## $ MCH : num [1:21] 30.3 28.9 29.6 29.6 30.3 31.5 30.1 32.2 30.5 31 ...
## $ MCHC : num [1:21] 33.3 33.2 34.2 34.6 34.9 34.9 34.1 34.3 33.5 34.4 ...
## $ MCV : num [1:21] 90.8 86.9 86.3 85.6 86.8 90.3 88.1 93.8 91 90 ...
## $ PLT : num [1:21] 177 255 236 219 240 198 181 232 223 173 ...

num_subjects <- nrow(Data_donors_pop)
print(num_subjects)

## [1] 21

dimensions <- dim(Data_donors_pop)
print(dimensions)

## [1] 21 17

age_histogram <- ggplot(Data_donors_pop, aes(x = Age)) +
 geom_histogram(aes(y = ..density..), bins = 10, color = "black", fill = "lightblue", alpha = 0.7) +
 stat_function(fun = dnorm, args = list(mean = mean(Data_donors_pop$Age), sd = sd(Data_donors_pop$Age)), color = "red") +
 geom_vline(xintercept = mean(Data_donors_pop$Age), color = "darkgreen", linetype = "dashed", size = 1) +
 geom_vline(xintercept = median(Data_donors_pop$Age), color = "blue", linetype = "dashed", size = 1) +
 geom_text(aes(x = mean(Data_donors_pop$Age), y = 0.02, label = paste("Mean: ", round(mean(Data_donors_pop$Age), 2))), vjust = -1.5, color = "black", size = 3) +
 geom_text(aes(x = median(Data_donors_pop$Age), y = 0.02, label = paste("Median: ", round(median(Data_donors_pop$Age), 2))), vjust = -0.5, color = "black", size = 3) +
 labs(title = "Age Distribution", x = "Age (years)", y = "Density") +
 theme_minimal()

### White Blood Cell (WBC) Distribution

wbc_histogram <- ggplot(Data_donors_pop, aes(x = WBC)) +
 geom_histogram(aes(y = ..density..), bins = 10, color = "black", fill = "lightgreen", alpha = 0.7) +
 stat_function(fun = dnorm, args = list(mean = mean(Data_donors_pop$WBC), sd = sd(Data_donors_pop$WBC)), color = "red") +
 geom_vline(xintercept = mean(Data_donors_pop$WBC), color = "darkgreen", linetype = "dashed", size = 1) +
 geom_vline(xintercept = median(Data_donors_pop$WBC), color = "blue", linetype = "dashed", size = 1) +
 geom_text(aes(x = mean(Data_donors_pop$WBC), y = 0.02, label = paste("Mean: ", round(mean(Data_donors_pop$WBC), 2))), vjust = -1.5, color = "black", size = 3) +
 geom_text(aes(x = median(Data_donors_pop$WBC), y = 0.02, label = paste("Median: ", round(median(Data_donors_pop$WBC), 2))), vjust = -0.5, color = "black", size = 3) +
 labs(title = "White Blood Cell (WBC) Distribution", x = "WBC (x10^3/µL)", y = "Density") +
 theme_minimal()

print(wbc_histogram)

### Lymphocyte Distribution

lymph_histogram <- ggplot(Data_donors_pop, aes(x = LYMPH)) +
 geom_histogram(aes(y = ..density..), bins = 10, color = "black", fill = "lightcoral", alpha = 0.7) +
 stat_function(fun = dnorm, args = list(mean = mean(Data_donors_pop$LYMPH), sd = sd(Data_donors_pop$LYMPH)), color = "red") +
 geom_vline(xintercept = mean(Data_donors_pop$LYMPH), color = "darkgreen", linetype = "dashed", size = 1) +
 geom_vline(xintercept = median(Data_donors_pop$LYMPH), color = "blue", linetype = "dashed", size = 1) +
 geom_text(aes(x = mean(Data_donors_pop$LYMPH), y = 0.02, label = paste("Mean: ", round(mean(Data_donors_pop$LYMPH), 2))), vjust = -1.5, color = "black", size = 3) +
 geom_text(aes(x = median(Data_donors_pop$LYMPH), y = 0.02, label = paste("Median: ", round(median(Data_donors_pop$LYMPH), 2))), vjust = -0.5, color = "black", size = 3) +
 labs(title = "Lymphocyte Distribution", x = "Lymphocytes (%)", y = "Density") +
 theme_minimal()

print(lymph_histogram)

### Correlation Analysis

correlation_coefficientW <- cor(Data_donors_pop$Age, Data_donors_pop$WBC)
print(paste("Correlation coefficient between Age and WBC:", round(correlation_coefficientW, 2)))

## [1] "Correlation coefficient between Age and WBC: -0.01"

ggplot(Data_donors_pop, aes(x = Age, y = LYMPH)) +
 geom_point(color = "blue", alpha = 0.7) +
 labs(title = "Scatter Plot of Age vs. Lymphocyte Percentage",
 x = "Age",
 y = "Lymphocyte (%)") +
 theme_minimal()

correlation_coefficientR <- cor(Data_donors_pop$Age, Data_donors_pop$LYMPH)
print(paste("Correlation coefficient between Age and Lymphocyte %:", round(correlation_coefficientR, 2)))

## [1] "Correlation coefficient between Age and Lymphocyte %: 0"

ggplot(Data_donors_pop, aes(x = WBC, y = LYMPH)) +
 geom_point(color = "blue", alpha = 0.7) +
 labs(title = "Scatter Plot of WBC vs. Lymphocyte Percentage",
 x = "WBC",
 y = "Lymphocyte (%)") +
 theme_minimal()

correlation_coefficientR <- cor(Data_donors_pop$WBC, Data_donors_pop$LYMPH)
print(paste("Correlation coefficient between WBC and Lymphocyte %:", round(correlation_coefficientR, 2)))

## [1] "Correlation coefficient between WBC and Lymphocyte %: -0.56"

## Luminex data – Statistical analysis in RStudio Version 4.3.1

library(readxl)
library(tidyverse)

library(ggplot2)
library(dplyr)
library(gridExtra)

library(MASS)

library(car)

# Set color schemes
colors_ir <- c("NoIR" = "#5B527a", "Photons" = "#FFb14e", "Fe" = "#FD0014")
colors_gravity <- c("1_g" = "#44AF69", "s_µ_g" = "#296B7A")
colors_th <- c("Th1" = "#FBB13C", "Th2" = "#0076A2", "Treg" = "#b8b8b8")

setwd("C:/Users/SFDSMIRA/Desktop/WP2 Data")
Data_donors <- read_excel("Master_file_results.xlsx", sheet = 7)

#Obtain infor about the data set
head(Data_donors)

## # A tibble: 6 × 16
## Cytokine Radiation Gravity Cortisol FIND001 FIND002 FIND005 FIND010 FIND012
## <chr> <chr> <chr> <dbl> <dbl> <dbl> <dbl> <dbl> <dbl>
## 1 IFN_G NoIR 1_g 0 100 100 100 100 100
## 2 TNF_A NoIR 1_g 0 100 100 100 100 100
## 3 IL_2 NoIR 1_g 0 100 100 100 100 100
## 4 IL_4 NoIR 1_g 0 100 100 100 100 100
## 5 IL_5 NoIR 1_g 0 100 100 100 100 100
## 6 IL_13 NoIR 1_g 0 100 100 100 100 100
## # ℹ 7 more variables: FIND013 <dbl>, FIND014 <dbl>, FIND015 <dbl>,
## # FIND017 <dbl>, FIND018 <dbl>, FIND025 <dbl>, FIND029 <dbl>

summary(Data_donors)

## Cytokine Radiation Gravity Cortisol
## Length:112 Length:112 Length:112 Min. :0.0
## Class :character Class :character Class :character 1st Qu.:0.0
## Mode :character Mode :character Mode :character Median :0.5
## Mean :0.5
## 3rd Qu.:1.0
## Max. :1.0
## FIND001 FIND002 FIND005 FIND010
## Min. : 0.1494 Min. : 0.128 Min. : 1.202 Min. : 0.3412
## 1st Qu.: 8.7643 1st Qu.: 12.002 1st Qu.: 20.504 1st Qu.: 7.2615
## Median : 27.4245 Median : 27.304 Median : 38.197 Median : 20.0406
## Mean : 47.8676 Mean : 66.002 Mean : 73.818 Mean : 39.2086
## 3rd Qu.: 82.7050 3rd Qu.:100.000 3rd Qu.:100.000 3rd Qu.: 54.1023
## Max. :289.0127 Max. :646.593 Max. :407.670 Max. :150.5027
## FIND012 FIND013 FIND014 FIND015
## Min. : 0.0058 Min. : 0.3351 Min. : 0.3446 Min. : 0.1592
## 1st Qu.: 2.4727 1st Qu.: 10.6589 1st Qu.: 14.5824 1st Qu.: 7.8176
## Median : 18.7408 Median : 33.5564 Median : 39.0134 Median : 15.0153
## Mean : 45.4213 Mean : 56.7421 Mean : 67.5986 Mean : 45.8141
## 3rd Qu.: 83.3889 3rd Qu.: 94.7796 3rd Qu.:100.0000 3rd Qu.: 59.8908
## Max. :464.0388 Max. :529.3755 Max. :559.8447 Max. :403.4685
## FIND017 FIND018 FIND025 FIND029
## Min. : 0.1681 Min. : 0.4705 Min. : 0.0263 Min. : 0.2563
## 1st Qu.: 6.8602 1st Qu.: 11.4044 1st Qu.: 6.8835 1st Qu.: 7.3441
## Median : 24.0502 Median : 24.5298 Median : 27.0662 Median : 22.3325
## Mean : 57.1338 Mean : 65.5271 Mean : 52.6230 Mean : 52.1263
## 3rd Qu.:100.0000 3rd Qu.:100.0000 3rd Qu.: 91.4171 3rd Qu.: 98.4390
## Max. :413.7421 Max. :611.1950 Max. :492.0997 Max. :435.8852

str(Data_donors)

## tibble [112 × 16] (S3: tbl_df/tbl/data.frame)
## $ Cytokine : chr [1:112] "IFN_G" "TNF_A" "IL_2" "IL_4" ...
## $ Radiation: chr [1:112] "NoIR" "NoIR" "NoIR" "NoIR" ...
## $ Gravity : chr [1:112] "1_g" "1_g" "1_g" "1_g" ...
## $ Cortisol : num [1:112] 0 0 0 0 0 0 0 1 1 1 ...
## $ FIND001 : num [1:112] 100 100 100 100 100 ...
## $ FIND002 : num [1:112] 100 100 100 100 100 ...
## $ FIND005 : num [1:112] 100 100 100 100 100 ...
## $ FIND010 : num [1:112] 100 100 100 100 100 ...
## $ FIND012 : num [1:112] 100 100 100 100 100 ...
## $ FIND013 : num [1:112] 100 100 100 100 100 ...
## $ FIND014 : num [1:112] 100 100 100 100 100 ...
## $ FIND015 : num [1:112] 100 100 100 100 100 ...
## $ FIND017 : num [1:112] 100 100 100 100 100 ...
## $ FIND018 : num [1:112] 100 100 100 100 100 ...
## $ FIND025 : num [1:112] 100 100 100 100 100 ...
## $ FIND029 : num [1:112] 100 100 100 100 100 ...

num_subjects <- nrow(Data_donors)
print(num_subjects)

## [1] 112

dimensions <- dim(Data_donors)
print(dimensions)

## [1] 112 16

library(ggplot2)

# Convert data to long format
Data_long <- tidyr::pivot_longer(Data_donors, cols = starts_with("FIND"), names_to = "Donor", values_to = "Cytokine_Level")
head(Data_long)

## # A tibble: 6 × 6
## Cytokine Radiation Gravity Cortisol Donor Cytokine_Level
## <chr> <chr> <chr> <dbl> <chr> <dbl>
## 1 IFN_G NoIR 1_g 0 FIND001 100
## 2 IFN_G NoIR 1_g 0 FIND002 100
## 3 IFN_G NoIR 1_g 0 FIND005 100
## 4 IFN_G NoIR 1_g 0 FIND010 100
## 5 IFN_G NoIR 1_g 0 FIND012 100
## 6 IFN_G NoIR 1_g 0 FIND013 100

# Convert Cortisol to factor with custom order
Data_long$Cortisol <- factor(Data_long$Cortisol, levels = c("0", "1"))

# Convert Gravity to factor with custom order
Data_long$Gravity <- factor(Data_long$Gravity, levels = c("1_g", "s_µ_g"))


# Convert Radiation to factor with custom order
Data_long$Radiation <- factor(Data_long$Radiation, levels = c("NoIR", "Photons", "Fe"))


# Filter data for cytokines
Data_IFN_G <- Data_long %>%
 filter(Cytokine == "IFN_G")
Data_TNF_A <- Data_long %>%
 filter(Cytokine == "TNF_A")
Data_IL_2 <- Data_long %>%
 filter(Cytokine == "IL_2")
Data_IL_4 <- Data_long %>%
 filter(Cytokine == "IL_4")
Data_IL_5 <- Data_long %>%
 filter(Cytokine == "IL_5")
Data_IL_13 <- Data_long %>%
 filter(Cytokine == "IL_13")
Data_IL_10 <- Data_long %>%
 filter(Cytokine == "IL_10")

# Define a function to remove outliers within each group based on cytokine levels
remove_outliers <- function(df) {
 df %>%
 group_by(Radiation, Cortisol, Gravity) %>%
 filter(across(starts_with("Cytokine_Level"), ~ between(., quantile(., 0.25) - 1.5 * IQR(.), quantile(., 0.75) + 1.5 * IQR(.)))) %>%
 ungroup()
}

# Remove outliers for each cytokine's data
Data_IFN_G_clean <- remove_outliers(Data_IFN_G)

Data_IL_2_clean <- remove_outliers(Data_IL_2)

Data_IL_5_clean <- remove_outliers(Data_IL_5)

Data_IL_13_clean <- remove_outliers(Data_IL_13)

Data_IL_10_clean <- remove_outliers(Data_IL_10)

# List of unique cytokines
cytokines <- unique(Data_long$Cytokine)


# Create dummy variables for Fe and Photons directly in Data_long
Data_long$Fe <- ifelse(Data_long$Radiation == "Fe", 1, 0)
Data_long$Photons <- ifelse(Data_long$Radiation == "Photons", 1, 0)

# Perform ANOVA for each cytokine
for(cytokine in cytokines) {
 cat("ANOVA for", cytokine, "\n")
 cytokine_data <- filter(Data_long, Cytokine == cytokine)
 lm_model <- lm(Cytokine_Level ~ Fe * Photons * Gravity * Cortisol, data = cytokine_data)
 anova_result <- anova(lm_model)
 print(anova_result)
 cat("\n")
}

## ANOVA for IFN_G
## Analysis of Variance Table
##
## Response: Cytokine_Level
## Df Sum Sq Mean Sq F value Pr(>F)
## Fe 1 39298 39298 50.7361 2.447e-11 ***
## Photons 1 566 566 0.7306 0.3938
## Gravity 1 241572 241572 311.8883 < 2.2e-16 ***
## Cortisol 1 95880 95880 123.7888 < 2.2e-16 ***
## Fe:Gravity 1 16329 16329 21.0823 8.239e-06 ***
## Photons:Gravity 1 582 582 0.7518 0.3871
## Fe:Cortisol 1 568 568 0.7338 0.3928
## Photons:Cortisol 1 193 193 0.2492 0.6183
## Gravity:Cortisol 1 81789 81789 105.5956 < 2.2e-16 ***
## Fe:Gravity:Cortisol 1 13 13 0.0172 0.8958
## Photons:Gravity:Cortisol 1 138 138 0.1776 0.6740
## Residuals 180 139419 775
## ---
## Signif. codes: 0 '***' 0.001 '**' 0.01 '*' 0.05 '.' 0.1 ' ' 1
##
## ANOVA for TNF_A
## Analysis of Variance Table
##
## Response: Cytokine_Level
## Df Sum Sq Mean Sq F value Pr(>F)
## Fe 1 252888 252888 365.3014 < 2.2e-16 ***
## Photons 1 716 716 1.0337 0.3107
## Gravity 1 360631 360631 520.9383 < 2.2e-16 ***
## Cortisol 1 168140 168140 242.8814 < 2.2e-16 ***
## Fe:Gravity 1 113895 113895 164.5234 < 2.2e-16 ***
## Photons:Gravity 1 633 633 0.9139 0.3404
## Fe:Cortisol 1 45311 45311 65.4520 8.549e-14 ***
## Photons:Cortisol 1 109 109 0.1572 0.6923
## Gravity:Cortisol 1 121302 121302 175.2232 < 2.2e-16 ***
## Fe:Gravity:Cortisol 1 26883 26883 38.8328 3.185e-09 ***
## Photons:Gravity:Cortisol 1 11 11 0.0160 0.8996
## Residuals 180 124609 692
## ---
## Signif. codes: 0 '***' 0.001 '**' 0.01 '*' 0.05 '.' 0.1 ' ' 1
##
## ANOVA for IL_2
## Analysis of Variance Table
##
## Response: Cytokine_Level
## Df Sum Sq Mean Sq F value Pr(>F)
## Fe 1 733321 733321 358.7400 < 2.2e-16 ***
## Photons 1 530 530 0.2592 0.6113
## Gravity 1 334154 334154 163.4679 < 2.2e-16 ***
## Cortisol 1 415976 415976 203.4954 < 2.2e-16 ***
## Fe:Gravity 1 185953 185953 90.9679 < 2.2e-16 ***
## Photons:Gravity 1 1190 1190 0.5824 0.4464
## Fe:Cortisol 1 258552 258552 126.4833 < 2.2e-16 ***
## Photons:Cortisol 1 1 1 0.0005 0.9830
## Gravity:Cortisol 1 135981 135981 66.5218 5.747e-14 ***
## Fe:Gravity:Cortisol 1 66648 66648 32.6040 4.588e-08 ***
## Photons:Gravity:Cortisol 1 48 48 0.0233 0.8790
## Residuals 180 367948 2044
## ---
## Signif. codes: 0 '***' 0.001 '**' 0.01 '*' 0.05 '.' 0.1 ' ' 1
##
## ANOVA for IL_4
## Analysis of Variance Table
##
## Response: Cytokine_Level
## Df Sum Sq Mean Sq F value Pr(>F)
## Fe 1 423580 423580 265.7830 < 2.2e-16 ***
## Photons 1 1466 1466 0.9198 0.33880
## Gravity 1 188290 188290 118.1458 < 2.2e-16 ***
## Cortisol 1 319218 319218 200.2989 < 2.2e-16 ***
## Fe:Gravity 1 25629 25629 16.0812 8.882e-05 ***
## Photons:Gravity 1 2533 2533 1.5892 0.20907
## Fe:Cortisol 1 130754 130754 82.0439 < 2.2e-16 ***
## Photons:Cortisol 1 399 399 0.2501 0.61765
## Gravity:Cortisol 1 92816 92816 58.2388 1.306e-12 ***
## Fe:Gravity:Cortisol 1 5863 5863 3.6787 0.05669 .
## Photons:Gravity:Cortisol 1 697 697 0.4375 0.50917
## Residuals 180 286867 1594
## ---
## Signif. codes: 0 '***' 0.001 '**' 0.01 '*' 0.05 '.' 0.1 ' ' 1
##
## ANOVA for IL_5
## Analysis of Variance Table
##
## Response: Cytokine_Level
## Df Sum Sq Mean Sq F value Pr(>F)
## Fe 1 55310 55310 105.6885 < 2.2e-16 ***
## Photons 1 0 0 0.0007 0.9792
## Gravity 1 103755 103755 198.2596 < 2.2e-16 ***
## Cortisol 1 146945 146945 280.7879 < 2.2e-16 ***
## Fe:Gravity 1 153 153 0.2928 0.5891
## Photons:Gravity 1 263 263 0.5016 0.4797
## Fe:Cortisol 1 19569 19569 37.3933 5.854e-09 ***
## Photons:Cortisol 1 13 13 0.0250 0.8745
## Gravity:Cortisol 1 75827 75827 144.8929 < 2.2e-16 ***
## Fe:Gravity:Cortisol 1 3 3 0.0053 0.9420
## Photons:Gravity:Cortisol 1 37 37 0.0713 0.7897
## Residuals 180 94200 523
## ---
## Signif. codes: 0 '***' 0.001 '**' 0.01 '*' 0.05 '.' 0.1 ' ' 1
##
## ANOVA for IL_13
## Analysis of Variance Table
##
## Response: Cytokine_Level
## Df Sum Sq Mean Sq F value Pr(>F)
## Fe 1 35730 35730 78.0146 9.008e-16 ***
## Photons 1 16 16 0.0354 0.8510691
## Gravity 1 109446 109446 238.9737 < 2.2e-16 ***
## Cortisol 1 54505 54505 119.0102 < 2.2e-16 ***
## Fe:Gravity 1 2600 2600 5.6766 0.0182343 *
## Photons:Gravity 1 61 61 0.1337 0.7150156
## Fe:Cortisol 1 6157 6157 13.4445 0.0003233 ***
## Photons:Cortisol 1 373 373 0.8143 0.3680604
## Gravity:Cortisol 1 42834 42834 93.5275 < 2.2e-16 ***
## Fe:Gravity:Cortisol 1 8 8 0.0184 0.8923267
## Photons:Gravity:Cortisol 1 35 35 0.0772 0.7814948
## Residuals 180 82437 458
## ---
## Signif. codes: 0 '***' 0.001 '**' 0.01 '*' 0.05 '.' 0.1 ' ' 1
##
## ANOVA for IL_10
## Analysis of Variance Table
##
## Response: Cytokine_Level
## Df Sum Sq Mean Sq F value Pr(>F)
## Fe 1 82935 82935 71.9271 7.941e-15 ***
## Photons 1 3063 3063 2.6568 0.104858
## Gravity 1 345850 345850 299.9467 < 2.2e-16 ***
## Cortisol 1 123185 123185 106.8354 < 2.2e-16 ***
## Fe:Gravity 1 56896 56896 49.3443 4.262e-11 ***
## Photons:Gravity 1 2272 2272 1.9703 0.162138
## Fe:Cortisol 1 10368 10368 8.9918 0.003096 **
## Photons:Cortisol 1 943 943 0.8181 0.366951
## Gravity:Cortisol 1 111411 111411 96.6242 < 2.2e-16 ***
## Fe:Gravity:Cortisol 1 8584 8584 7.4445 0.006994 **
## Photons:Gravity:Cortisol 1 684 684 0.5929 0.442292
## Residuals 180 207547 1153
## ---
## Signif. codes: 0 '***' 0.001 '**' 0.01 '*' 0.05 '.' 0.1 ' ' 1

# Calculate quartiles, IQR, and identify outliers grouped by conditions
outliers <- Data_long %>%
 group_by(Radiation, Cortisol, Gravity) %>%
 mutate(
 Q1 = quantile(Cytokine_Level, 0.25),
 Q3 = quantile(Cytokine_Level, 0.75),
 IQR = Q3 - Q1,
 lower_bound = Q1 - 1.5 * IQR,
 upper_bound = Q3 + 1.5 * IQR,
 outlier = Cytokine_Level < lower_bound | Cytokine_Level > upper_bound
 ) %>%
 ungroup()

# Remove outliers
Data_no_outliers <- outliers %>%
 filter(!outlier)

# Check the impact on ANOVA results
# Perform ANOVA for each cytokine with and without outliers
for(cytokine in cytokines) {
 cat("ANOVA for", cytokine, "\n")
 cytokine_data_no_outlier <- filter(Data_no_outliers, Cytokine == cytokine)
 lm_model_noout <- lm(Cytokine_Level ~ Fe * Photons * Gravity * Cortisol, data = cytokine_data_no_outlier)

 # Print the summary of the linear regression model
 print(summary(lm_model_noout))

 # Print ANOVA result
 anova_result <- anova(lm_model_noout)
 print(anova_result)
 cat("\n")
}

## ANOVA for IFN_G
##
## Call:
## lm(formula = Cytokine_Level ~ Fe * Photons * Gravity * Cortisol,
## data = cytokine_data_no_outlier)
##
## Residuals:
## Min 1Q Median 3Q Max
## -129.491 -2.612 -0.356 1.203 166.330
##
## Coefficients: (4 not defined because of singularities)
## Estimate Std. Error t value Pr(>|t|)
## (Intercept) 100.000 4.974 20.106 < 2e-16 ***
## Fe 63.252 8.615 7.342 7.47e-12 ***
## Photons -1.944 9.524 -0.204 0.838509
## Gravitys_µ_g -97.042 7.034 -13.797 < 2e-16 ***
## Cortisol1 -82.548 7.110 -11.610 < 2e-16 ***
## Fe:Photons NA NA NA NA
## Fe:Gravitys_µ_g -48.038 12.183 -3.943 0.000116 ***
## Photons:Gravitys_µ_g 2.266 12.842 0.176 0.860140
## Fe:Cortisol1 -10.298 12.227 -0.842 0.400817
## Photons:Cortisol1 7.771 12.884 0.603 0.547153
## Gravitys_µ_g:Cortisol1 81.070 10.001 8.106 8.52e-14 ***
## Fe:Photons:Gravitys_µ_g NA NA NA NA
## Fe:Photons:Cortisol1 NA NA NA NA
## Fe:Gravitys_µ_g:Cortisol1 3.313 17.260 0.192 0.848028
## Photons:Gravitys_µ_g:Cortisol1 -8.537 17.732 -0.481 0.630789
## Fe:Photons:Gravitys_µ_g:Cortisol1 NA NA NA NA
## ---
## Signif. codes: 0 '***' 0.001 '**' 0.01 '*' 0.05 '.' 0.1 ' ' 1
##
## Residual standard error: 24.37 on 176 degrees of freedom
## Multiple R-squared: 0.8097, Adjusted R-squared: 0.7978
## F-statistic: 68.06 on 11 and 176 DF, p-value: < 2.2e-16
##
## Analysis of Variance Table
##
## Response: Cytokine_Level
## Df Sum Sq Mean Sq F value Pr(>F)
## Fe 1 46184 46184 77.7921 1.099e-15 ***
## Photons 1 404 404 0.6812 0.4103
## Gravity 1 217146 217146 365.7599 < 2.2e-16 ***
## Cortisol 1 85407 85407 143.8590 < 2.2e-16 ***
## Fe:Gravity 1 19057 19057 32.0988 5.880e-08 ***
## Photons:Gravity 1 4 4 0.0068 0.9344
## Fe:Cortisol 1 1070 1070 1.8025 0.1811
## Photons:Cortisol 1 260 260 0.4382 0.5089
## Gravity:Cortisol 1 74723 74723 125.8621 < 2.2e-16 ***
## Fe:Gravity:Cortisol 1 81 81 0.1366 0.7122
## Photons:Gravity:Cortisol 1 138 138 0.2318 0.6308
## Residuals 176 104489 594
## ---
## Signif. codes: 0 '***' 0.001 '**' 0.01 '*' 0.05 '.' 0.1 ' ' 1
##
## ANOVA for TNF_A
##
## Call:
## lm(formula = Cytokine_Level ~ Fe * Photons * Gravity * Cortisol,
## data = cytokine_data_no_outlier)
##
## Residuals:
## Min 1Q Median 3Q Max
## -174.372 -4.427 0.000 2.772 111.492
##
## Coefficients: (4 not defined because of singularities)
## Estimate Std. Error t value Pr(>|t|)
## (Intercept) 100.000 4.910 20.368 < 2e-16 ***
## Fe 206.733 8.504 24.311 < 2e-16 ***
## Photons 11.607 8.504 1.365 0.174
## Gravitys_µ_g -91.806 6.943 -13.222 < 2e-16 ***
## Cortisol1 -76.433 6.943 -11.008 < 2e-16 ***
## Fe:Photons NA NA NA NA
## Fe:Gravitys_µ_g -170.504 12.026 -14.178 < 2e-16 ***
## Photons:Gravitys_µ_g -10.068 12.026 -0.837 0.404
## Fe:Cortisol1 -139.881 12.207 -11.459 < 2e-16 ***
## Photons:Cortisol1 -4.863 12.026 -0.404 0.686
## Gravitys_µ_g:Cortisol1 71.474 9.872 7.240 1.30e-11 ***
## Fe:Photons:Gravitys_µ_g NA NA NA NA
## Fe:Photons:Cortisol1 NA NA NA NA
## Fe:Gravitys_µ_g:Cortisol1 123.699 17.166 7.206 1.58e-11 ***
## Photons:Gravitys_µ_g:Cortisol1 3.307 17.038 0.194 0.846
## Fe:Photons:Gravitys_µ_g:Cortisol1 NA NA NA NA
## ---
## Signif. codes: 0 '***' 0.001 '**' 0.01 '*' 0.05 '.' 0.1 ' ' 1
##
## Residual standard error: 24.05 on 178 degrees of freedom
## Multiple R-squared: 0.9127, Adjusted R-squared: 0.9073
## F-statistic: 169.2 on 11 and 178 DF, p-value: < 2.2e-16
##
## Analysis of Variance Table
##
## Response: Cytokine_Level
## Df Sum Sq Mean Sq F value Pr(>F)
## Fe 1 232852 232852 402.5138 < 2.2e-16 ***
## Photons 1 689 689 1.1903 0.2767
## Gravity 1 352899 352899 610.0291 < 2.2e-16 ***
## Cortisol 1 177018 177018 305.9980 < 2.2e-16 ***
## Fe:Gravity 1 104678 104678 180.9485 < 2.2e-16 ***
## Photons:Gravity 1 488 488 0.8436 0.3596
## Fe:Cortisol 1 49087 49087 84.8527 < 2.2e-16 ***
## Photons:Cortisol 1 64 64 0.1100 0.7406
## Gravity:Cortisol 1 125628 125628 217.1629 < 2.2e-16 ***
## Fe:Gravity:Cortisol 1 33188 33188 57.3689 1.893e-12 ***
## Photons:Gravity:Cortisol 1 22 22 0.0377 0.8463
## Residuals 178 102972 578
## ---
## Signif. codes: 0 '***' 0.001 '**' 0.01 '*' 0.05 '.' 0.1 ' ' 1
##
## ANOVA for IL_2
##
## Call:
## lm(formula = Cytokine_Level ~ Fe * Photons * Gravity * Cortisol,
## data = cytokine_data_no_outlier)
##
## Residuals:
## Min 1Q Median 3Q Max
## -272.883 -4.706 0.000 3.978 150.296
##
## Coefficients: (4 not defined because of singularities)
## Estimate Std. Error t value Pr(>|t|)
## (Intercept) 100.000 6.927 14.436 < 2e-16 ***
## Fe 309.549 12.773 24.234 < 2e-16 ***
## Photons 11.216 11.998 0.935 0.351
## Gravitys_µ_g -74.334 9.797 -7.588 1.93e-12 ***
## Cortisol1 -81.732 9.797 -8.343 2.21e-14 ***
## Fe:Photons NA NA NA NA
## Fe:Gravitys_µ_g -220.845 18.064 -12.225 < 2e-16 ***
## Photons:Gravitys_µ_g -14.636 16.968 -0.863 0.390
## Fe:Cortisol1 -239.716 17.772 -13.488 < 2e-16 ***
## Photons:Cortisol1 -2.096 16.968 -0.124 0.902
## Gravitys_µ_g:Cortisol1 60.618 13.930 4.352 2.30e-05 ***
## Fe:Photons:Gravitys_µ_g NA NA NA NA
## Fe:Photons:Cortisol1 NA NA NA NA
## Fe:Gravitys_µ_g:Cortisol1 176.233 25.175 7.000 5.40e-11 ***
## Photons:Gravitys_µ_g:Cortisol1 6.056 24.040 0.252 0.801
## Fe:Photons:Gravitys_µ_g:Cortisol1 NA NA NA NA
## ---
## Signif. codes: 0 '***' 0.001 '**' 0.01 '*' 0.05 '.' 0.1 ' ' 1
##
## Residual standard error: 33.94 on 173 degrees of freedom
## Multiple R-squared: 0.8855, Adjusted R-squared: 0.8783
## F-statistic: 121.7 on 11 and 173 DF, p-value: < 2.2e-16
##
## Analysis of Variance Table
##
## Response: Cytokine_Level
## Df Sum Sq Mean Sq F value Pr(>F)
## Fe 1 442895 442895 384.5550 < 2.2e-16 ***
## Photons 1 516 516 0.4477 0.5043
## Gravity 1 265550 265550 230.5703 < 2.2e-16 ***
## Cortisol 1 340919 340919 296.0111 < 2.2e-16 ***
## Fe:Gravity 1 122260 122260 106.1550 < 2.2e-16 ***
## Photons:Gravity 1 951 951 0.8255 0.3648
## Fe:Cortisol 1 186434 186434 161.8755 < 2.2e-16 ***
## Photons:Cortisol 1 13 13 0.0109 0.9168
## Gravity:Cortisol 1 120536 120536 104.6588 < 2.2e-16 ***
## Fe:Gravity:Cortisol 1 61461 61461 53.3653 9.734e-12 ***
## Photons:Gravity:Cortisol 1 73 73 0.0635 0.8014
## Residuals 173 199246 1152
## ---
## Signif. codes: 0 '***' 0.001 '**' 0.01 '*' 0.05 '.' 0.1 ' ' 1
##
## ANOVA for IL_4
##
## Call:
## lm(formula = Cytokine_Level ~ Fe * Photons * Gravity * Cortisol,
## data = cytokine_data_no_outlier)
##
## Residuals:
## Min 1Q Median 3Q Max
## -174.414 -6.764 0.000 5.360 237.598
##
## Coefficients: (4 not defined because of singularities)
## Estimate Std. Error t value Pr(>|t|)
## (Intercept) 100.000 7.020 14.244 < 2e-16 ***
## Fe 216.135 12.160 17.775 < 2e-16 ***
## Photons 23.862 12.160 1.962 0.051312 .
## Gravitys_µ_g -77.840 9.928 -7.840 4.32e-13 ***
## Cortisol1 -83.545 9.928 -8.415 1.39e-14 ***
## Fe:Photons NA NA NA NA
## Fe:Gravitys_µ_g -131.183 18.126 -7.237 1.41e-11 ***
## Photons:Gravitys_µ_g -27.129 17.196 -1.578 0.116473
## Fe:Cortisol1 -151.521 17.196 -8.811 1.23e-15 ***
## Photons:Cortisol1 -16.394 17.196 -0.953 0.341744
## Gravitys_µ_g:Cortisol1 66.829 14.199 4.706 5.12e-06 ***
## Fe:Photons:Gravitys_µ_g NA NA NA NA
## Fe:Photons:Cortisol1 NA NA NA NA
## Fe:Gravitys_µ_g:Cortisol1 95.704 25.253 3.790 0.000208 ***
## Photons:Gravitys_µ_g:Cortisol1 20.804 24.411 0.852 0.395250
## Fe:Photons:Gravitys_µ_g:Cortisol1 NA NA NA NA
## ---
## Signif. codes: 0 '***' 0.001 '**' 0.01 '*' 0.05 '.' 0.1 ' ' 1
##
## Residual standard error: 34.39 on 174 degrees of freedom
## Multiple R-squared: 0.8449, Adjusted R-squared: 0.8351
## F-statistic: 86.18 on 11 and 174 DF, p-value: < 2.2e-16
##
## Analysis of Variance Table
##
## Response: Cytokine_Level
## Df Sum Sq Mean Sq F value Pr(>F)
## Fe 1 335708 335708 283.8161 < 2.2e-16 ***
## Photons 1 1406 1406 1.1883 0.2771703
## Gravity 1 220988 220988 186.8290 < 2.2e-16 ***
## Cortisol 1 292086 292086 246.9368 < 2.2e-16 ***
## Fe:Gravity 1 48789 48789 41.2473 1.236e-09 ***
## Photons:Gravity 1 1880 1880 1.5896 0.2090649
## Fe:Cortisol 1 99311 99311 83.9597 < 2.2e-16 ***
## Photons:Cortisol 1 219 219 0.1850 0.6676615
## Gravity:Cortisol 1 103704 103704 87.6737 < 2.2e-16 ***
## Fe:Gravity:Cortisol 1 16328 16328 13.8041 0.0002732 ***
## Photons:Gravity:Cortisol 1 859 859 0.7263 0.3952498
## Residuals 174 205814 1183
## ---
## Signif. codes: 0 '***' 0.001 '**' 0.01 '*' 0.05 '.' 0.1 ' ' 1
##
## ANOVA for IL_5
##
## Call:
## lm(formula = Cytokine_Level ~ Fe * Photons * Gravity * Cortisol,
## data = cytokine_data_no_outlier)
##
## Residuals:
## Min 1Q Median 3Q Max
## -73.347 -6.757 -0.157 4.982 105.141
##
## Coefficients: (4 not defined because of singularities)
## Estimate Std. Error t value Pr(>|t|)
## (Intercept) 100.000 4.170 23.981 < 2e-16 ***
## Fe 66.346 7.222 9.186 < 2e-16 ***
## Photons 4.478 7.222 0.620 0.536
## Gravitys_µ_g -82.438 5.897 -13.979 < 2e-16 ***
## Cortisol1 -83.766 5.961 -14.053 < 2e-16 ***
## Fe:Photons NA NA NA NA
## Fe:Gravitys_µ_g -7.312 10.214 -0.716 0.475
## Photons:Gravitys_µ_g -7.889 10.214 -0.772 0.441
## Fe:Cortisol1 -46.699 10.251 -4.555 9.74e-06 ***
## Photons:Cortisol1 -1.806 10.251 -0.176 0.860
## Gravitys_µ_g:Cortisol1 76.308 8.479 9.000 3.59e-16 ***
## Fe:Photons:Gravitys_µ_g NA NA NA NA
## Fe:Photons:Cortisol1 NA NA NA NA
## Fe:Gravitys_µ_g:Cortisol1 -6.983 14.634 -0.477 0.634
## Photons:Gravitys_µ_g:Cortisol1 5.785 14.526 0.398 0.691
## Fe:Photons:Gravitys_µ_g:Cortisol1 NA NA NA NA
## ---
## Signif. codes: 0 '***' 0.001 '**' 0.01 '*' 0.05 '.' 0.1 ' ' 1
##
## Residual standard error: 20.43 on 176 degrees of freedom
## Multiple R-squared: 0.8486, Adjusted R-squared: 0.8391
## F-statistic: 89.67 on 11 and 176 DF, p-value: < 2.2e-16
##
## Analysis of Variance Table
##
## Response: Cytokine_Level
## Df Sum Sq Mean Sq F value Pr(>F)
## Fe 1 50787 50787 121.7000 < 2.2e-16 ***
## Photons 1 3 3 0.0072 0.9324
## Gravity 1 109575 109575 262.5724 < 2.2e-16 ***
## Cortisol 1 159485 159485 382.1717 < 2.2e-16 ***
## Fe:Gravity 1 583 583 1.3963 0.2389
## Photons:Gravity 1 104 104 0.2485 0.6188
## Fe:Cortisol 1 22939 22939 54.9680 4.959e-12 ***
## Photons:Cortisol 1 18 18 0.0441 0.8340
## Gravity:Cortisol 1 67876 67876 162.6489 < 2.2e-16 ***
## Fe:Gravity:Cortisol 1 176 176 0.4228 0.5164
## Photons:Gravity:Cortisol 1 66 66 0.1586 0.6909
## Residuals 176 73447 417
## ---
## Signif. codes: 0 '***' 0.001 '**' 0.01 '*' 0.05 '.' 0.1 ' ' 1
##
## ANOVA for IL_13
##
## Call:
## lm(formula = Cytokine_Level ~ Fe * Photons * Gravity * Cortisol,
## data = cytokine_data_no_outlier)
##
## Residuals:
## Min 1Q Median 3Q Max
## -63.401 -6.955 0.000 8.212 68.074
##
## Coefficients: (4 not defined because of singularities)
## Estimate Std. Error t value Pr(>|t|)
## (Intercept) 100.000 3.601 27.768 < 2e-16 ***
## Fe 54.575 6.237 8.750 5.88e-15 ***
## Photons 3.035 6.237 0.487 0.6273
## Gravitys_µ_g -80.579 5.341 -15.086 < 2e-16 ***
## Cortisol1 -63.937 5.694 -11.229 < 2e-16 ***
## Fe:Photons NA NA NA NA
## Fe:Gravitys_µ_g -10.517 8.967 -1.173 0.2428
## Photons:Gravitys_µ_g 3.382 9.097 0.372 0.7106
## Fe:Cortisol1 -19.976 9.181 -2.176 0.0312 *
## Photons:Cortisol1 3.997 9.181 0.435 0.6640
## Gravitys_µ_g:Cortisol1 52.562 9.322 5.639 9.03e-08 ***
## Fe:Photons:Gravitys_µ_g NA NA NA NA
## Fe:Photons:Cortisol1 NA NA NA NA
## Fe:Gravitys_µ_g:Cortisol1 4.207 13.892 0.303 0.7625
## Photons:Gravitys_µ_g:Cortisol1 -7.813 16.456 -0.475 0.6357
## Fe:Photons:Gravitys_µ_g:Cortisol1 NA NA NA NA
## ---
## Signif. codes: 0 '***' 0.001 '**' 0.01 '*' 0.05 '.' 0.1 ' ' 1
##
## Residual standard error: 17.64 on 141 degrees of freedom
## Multiple R-squared: 0.8568, Adjusted R-squared: 0.8457
## F-statistic: 76.72 on 11 and 141 DF, p-value: < 2.2e-16
##
## Analysis of Variance Table
##
## Response: Cytokine_Level
## Df Sum Sq Mean Sq F value Pr(>F)
## Fe 1 29968 29968 96.2814 < 2.2e-16 ***
## Photons 1 396 396 1.2709 0.26150
## Gravity 1 123057 123057 395.3626 < 2.2e-16 ***
## Cortisol 1 85549 85549 274.8571 < 2.2e-16 ***
## Fe:Gravity 1 6 6 0.0197 0.88844
## Photons:Gravity 1 2 2 0.0071 0.93310
## Fe:Cortisol 1 912 912 2.9286 0.08922 .
## Photons:Cortisol 1 48 48 0.1557 0.69372
## Gravity:Cortisol 1 22583 22583 72.5560 2.211e-14 ***
## Fe:Gravity:Cortisol 1 85 85 0.2730 0.60217
## Photons:Gravity:Cortisol 1 70 70 0.2254 0.63569
## Residuals 141 43886 311
## ---
## Signif. codes: 0 '***' 0.001 '**' 0.01 '*' 0.05 '.' 0.1 ' ' 1
##
## ANOVA for IL_10
##
## Call:
## lm(formula = Cytokine_Level ~ Fe * Photons * Gravity * Cortisol,
## data = cytokine_data_no_outlier)
##
## Residuals:
## Min 1Q Median 3Q Max
## -88.351 -4.312 -1.018 0.000 173.884
##
## Coefficients: (4 not defined because of singularities)
## Estimate Std. Error t value Pr(>|t|)
## (Intercept) 100.000 4.411 22.671 < 2e-16 ***
## Fe 129.584 7.640 16.961 < 2e-16 ***
## Photons 4.787 7.868 0.608 0.544
## Gravitys_µ_g -96.764 6.238 -15.512 < 2e-16 ***
## Cortisol1 -75.930 6.238 -12.172 < 2e-16 ***
## Fe:Photons NA NA NA NA
## Fe:Gravitys_µ_g -119.091 10.805 -11.022 < 2e-16 ***
## Photons:Gravitys_µ_g -2.621 10.967 -0.239 0.811
## Fe:Cortisol1 -91.330 10.967 -8.328 2.19e-14 ***
## Photons:Cortisol1 3.372 10.967 0.307 0.759
## Gravitys_µ_g:Cortisol1 73.927 8.870 8.335 2.10e-14 ***
## Fe:Photons:Gravitys_µ_g NA NA NA NA
## Fe:Photons:Cortisol1 NA NA NA NA
## Fe:Gravitys_µ_g:Cortisol1 88.557 15.423 5.742 4.00e-08 ***
## Photons:Gravitys_µ_g:Cortisol1 -4.163 15.423 -0.270 0.788
## Fe:Photons:Gravitys_µ_g:Cortisol1 NA NA NA NA
## ---
## Signif. codes: 0 '***' 0.001 '**' 0.01 '*' 0.05 '.' 0.1 ' ' 1
##
## Residual standard error: 21.61 on 177 degrees of freedom
## Multiple R-squared: 0.893, Adjusted R-squared: 0.8864
## F-statistic: 134.4 on 11 and 177 DF, p-value: < 2.2e-16
##
## Analysis of Variance Table
##
## Response: Cytokine_Level
## Df Sum Sq Mean Sq F value Pr(>F)
## Fe 1 73947 73947 158.3563 < 2.2e-16 ***
## Photons 1 172 172 0.3680 0.5449
## Gravity 1 302858 302858 648.5621 < 2.2e-16 ***
## Cortisol 1 119998 119998 256.9724 < 2.2e-16 ***
## Fe:Gravity 1 50463 50463 108.0656 < 2.2e-16 ***
## Photons:Gravity 1 108 108 0.2304 0.6318
## Fe:Cortisol 1 18393 18393 39.3882 2.597e-09 ***
## Photons:Cortisol 1 48 48 0.1021 0.7498
## Gravity:Cortisol 1 106297 106297 227.6320 < 2.2e-16 ***
## Fe:Gravity:Cortisol 1 17828 17828 38.1791 4.316e-09 ***
## Photons:Gravity:Cortisol 1 34 34 0.0729 0.7875
## Residuals 177 82653 467
## ---
## Signif. codes: 0 '***' 0.001 '**' 0.01 '*' 0.05 '.' 0.1 ' ' 1

plot(lm_model_noout)

### Goodness of fit

# Create a vector to store AIC values for each cytokine
aic_values <- numeric(length(cytokines))

# Fit linear regression models for each cytokine
for(cytokine in cytokines) {
 cat("ANOVA for", cytokine, "\n")
 cytokine_data_no_outlier <- filter(Data_no_outliers, Cytokine == cytokine)
 lm_model_noout <- lm(Cytokine_Level ~ Fe * Photons * Gravity * Cortisol, data = cytokine_data_no_outlier)

 # Print the summary of the linear regression model
 print(summary(lm_model_noout))

 # Calculate AIC for the model
 aic_values[cytokine] <- AIC(lm_model_noout)

 # Print ANOVA result
 anova_result <- anova(lm_model_noout)
 print(anova_result)
 cat("\n")
}

## ANOVA for IFN_G
##
## Call:
## lm(formula = Cytokine_Level ~ Fe * Photons * Gravity * Cortisol,
## data = cytokine_data_no_outlier)
##
## Residuals:
## Min 1Q Median 3Q Max
## -129.491 -2.612 -0.356 1.203 166.330
##
## Coefficients: (4 not defined because of singularities)
## Estimate Std. Error t value Pr(>|t|)
## (Intercept) 100.000 4.974 20.106 < 2e-16 ***
## Fe 63.252 8.615 7.342 7.47e-12 ***
## Photons -1.944 9.524 -0.204 0.838509
## Gravitys_µ_g -97.042 7.034 -13.797 < 2e-16 ***
## Cortisol1 -82.548 7.110 -11.610 < 2e-16 ***
## Fe:Photons NA NA NA NA
## Fe:Gravitys_µ_g -48.038 12.183 -3.943 0.000116 ***
## Photons:Gravitys_µ_g 2.266 12.842 0.176 0.860140
## Fe:Cortisol1 -10.298 12.227 -0.842 0.400817
## Photons:Cortisol1 7.771 12.884 0.603 0.547153
## Gravitys_µ_g:Cortisol1 81.070 10.001 8.106 8.52e-14 ***
## Fe:Photons:Gravitys_µ_g NA NA NA NA
## Fe:Photons:Cortisol1 NA NA NA NA
## Fe:Gravitys_µ_g:Cortisol1 3.313 17.260 0.192 0.848028
## Photons:Gravitys_µ_g:Cortisol1 -8.537 17.732 -0.481 0.630789
## Fe:Photons:Gravitys_µ_g:Cortisol1 NA NA NA NA
## ---
## Signif. codes: 0 '***' 0.001 '**' 0.01 '*' 0.05 '.' 0.1 ' ' 1
##
## Residual standard error: 24.37 on 176 degrees of freedom
## Multiple R-squared: 0.8097, Adjusted R-squared: 0.7978
## F-statistic: 68.06 on 11 and 176 DF, p-value: < 2.2e-16
##
## Analysis of Variance Table
##
## Response: Cytokine_Level
## Df Sum Sq Mean Sq F value Pr(>F)
## Fe 1 46184 46184 77.7921 1.099e-15 ***
## Photons 1 404 404 0.6812 0.4103
## Gravity 1 217146 217146 365.7599 < 2.2e-16 ***
## Cortisol 1 85407 85407 143.8590 < 2.2e-16 ***
## Fe:Gravity 1 19057 19057 32.0988 5.880e-08 ***
## Photons:Gravity 1 4 4 0.0068 0.9344
## Fe:Cortisol 1 1070 1070 1.8025 0.1811
## Photons:Cortisol 1 260 260 0.4382 0.5089
## Gravity:Cortisol 1 74723 74723 125.8621 < 2.2e-16 ***
## Fe:Gravity:Cortisol 1 81 81 0.1366 0.7122
## Photons:Gravity:Cortisol 1 138 138 0.2318 0.6308
## Residuals 176 104489 594
## ---
## Signif. codes: 0 '***' 0.001 '**' 0.01 '*' 0.05 '.' 0.1 ' ' 1
##
## ANOVA for TNF_A
##
## Call:
## lm(formula = Cytokine_Level ~ Fe * Photons * Gravity * Cortisol,
## data = cytokine_data_no_outlier)
##
## Residuals:
## Min 1Q Median 3Q Max
## -174.372 -4.427 0.000 2.772 111.492
##
## Coefficients: (4 not defined because of singularities)
## Estimate Std. Error t value Pr(>|t|)
## (Intercept) 100.000 4.910 20.368 < 2e-16 ***
## Fe 206.733 8.504 24.311 < 2e-16 ***
## Photons 11.607 8.504 1.365 0.174
## Gravitys_µ_g -91.806 6.943 -13.222 < 2e-16 ***
## Cortisol1 -76.433 6.943 -11.008 < 2e-16 ***
## Fe:Photons NA NA NA NA
## Fe:Gravitys_µ_g -170.504 12.026 -14.178 < 2e-16 ***
## Photons:Gravitys_µ_g -10.068 12.026 -0.837 0.404
## Fe:Cortisol1 -139.881 12.207 -11.459 < 2e-16 ***
## Photons:Cortisol1 -4.863 12.026 -0.404 0.686
## Gravitys_µ_g:Cortisol1 71.474 9.872 7.240 1.30e-11 ***
## Fe:Photons:Gravitys_µ_g NA NA NA NA
## Fe:Photons:Cortisol1 NA NA NA NA
## Fe:Gravitys_µ_g:Cortisol1 123.699 17.166 7.206 1.58e-11 ***
## Photons:Gravitys_µ_g:Cortisol1 3.307 17.038 0.194 0.846
## Fe:Photons:Gravitys_µ_g:Cortisol1 NA NA NA NA
## ---
## Signif. codes: 0 '***' 0.001 '**' 0.01 '*' 0.05 '.' 0.1 ' ' 1
##
## Residual standard error: 24.05 on 178 degrees of freedom
## Multiple R-squared: 0.9127, Adjusted R-squared: 0.9073
## F-statistic: 169.2 on 11 and 178 DF, p-value: < 2.2e-16
##
## Analysis of Variance Table
##
## Response: Cytokine_Level
## Df Sum Sq Mean Sq F value Pr(>F)
## Fe 1 232852 232852 402.5138 < 2.2e-16 ***
## Photons 1 689 689 1.1903 0.2767
## Gravity 1 352899 352899 610.0291 < 2.2e-16 ***
## Cortisol 1 177018 177018 305.9980 < 2.2e-16 ***
## Fe:Gravity 1 104678 104678 180.9485 < 2.2e-16 ***
## Photons:Gravity 1 488 488 0.8436 0.3596
## Fe:Cortisol 1 49087 49087 84.8527 < 2.2e-16 ***
## Photons:Cortisol 1 64 64 0.1100 0.7406
## Gravity:Cortisol 1 125628 125628 217.1629 < 2.2e-16 ***
## Fe:Gravity:Cortisol 1 33188 33188 57.3689 1.893e-12 ***
## Photons:Gravity:Cortisol 1 22 22 0.0377 0.8463
## Residuals 178 102972 578
## ---
## Signif. codes: 0 '***' 0.001 '**' 0.01 '*' 0.05 '.' 0.1 ' ' 1
##
## ANOVA for IL_2
##
## Call:
## lm(formula = Cytokine_Level ~ Fe * Photons * Gravity * Cortisol,
## data = cytokine_data_no_outlier)
##
## Residuals:
## Min 1Q Median 3Q Max
## -272.883 -4.706 0.000 3.978 150.296
##
## Coefficients: (4 not defined because of singularities)
## Estimate Std. Error t value Pr(>|t|)
## (Intercept) 100.000 6.927 14.436 < 2e-16 ***
## Fe 309.549 12.773 24.234 < 2e-16 ***
## Photons 11.216 11.998 0.935 0.351
## Gravitys_µ_g -74.334 9.797 -7.588 1.93e-12 ***
## Cortisol1 -81.732 9.797 -8.343 2.21e-14 ***
## Fe:Photons NA NA NA NA
## Fe:Gravitys_µ_g -220.845 18.064 -12.225 < 2e-16 ***
## Photons:Gravitys_µ_g -14.636 16.968 -0.863 0.390
## Fe:Cortisol1 -239.716 17.772 -13.488 < 2e-16 ***
## Photons:Cortisol1 -2.096 16.968 -0.124 0.902
## Gravitys_µ_g:Cortisol1 60.618 13.930 4.352 2.30e-05 ***
## Fe:Photons:Gravitys_µ_g NA NA NA NA
## Fe:Photons:Cortisol1 NA NA NA NA
## Fe:Gravitys_µ_g:Cortisol1 176.233 25.175 7.000 5.40e-11 ***
## Photons:Gravitys_µ_g:Cortisol1 6.056 24.040 0.252 0.801
## Fe:Photons:Gravitys_µ_g:Cortisol1 NA NA NA NA
## ---
## Signif. codes: 0 '***' 0.001 '**' 0.01 '*' 0.05 '.' 0.1 ' ' 1
##
## Residual standard error: 33.94 on 173 degrees of freedom
## Multiple R-squared: 0.8855, Adjusted R-squared: 0.8783
## F-statistic: 121.7 on 11 and 173 DF, p-value: < 2.2e-16
##
## Analysis of Variance Table
##
## Response: Cytokine_Level
## Df Sum Sq Mean Sq F value Pr(>F)
## Fe 1 442895 442895 384.5550 < 2.2e-16 ***
## Photons 1 516 516 0.4477 0.5043
## Gravity 1 265550 265550 230.5703 < 2.2e-16 ***
## Cortisol 1 340919 340919 296.0111 < 2.2e-16 ***
## Fe:Gravity 1 122260 122260 106.1550 < 2.2e-16 ***
## Photons:Gravity 1 951 951 0.8255 0.3648
## Fe:Cortisol 1 186434 186434 161.8755 < 2.2e-16 ***
## Photons:Cortisol 1 13 13 0.0109 0.9168
## Gravity:Cortisol 1 120536 120536 104.6588 < 2.2e-16 ***
## Fe:Gravity:Cortisol 1 61461 61461 53.3653 9.734e-12 ***
## Photons:Gravity:Cortisol 1 73 73 0.0635 0.8014
## Residuals 173 199246 1152
## ---
## Signif. codes: 0 '***' 0.001 '**' 0.01 '*' 0.05 '.' 0.1 ' ' 1
##
## ANOVA for IL_4
##
## Call:
## lm(formula = Cytokine_Level ~ Fe * Photons * Gravity * Cortisol,
## data = cytokine_data_no_outlier)
##
## Residuals:
## Min 1Q Median 3Q Max
## -174.414 -6.764 0.000 5.360 237.598
##
## Coefficients: (4 not defined because of singularities)
## Estimate Std. Error t value Pr(>|t|)
## (Intercept) 100.000 7.020 14.244 < 2e-16 ***
## Fe 216.135 12.160 17.775 < 2e-16 ***
## Photons 23.862 12.160 1.962 0.051312 .
## Gravitys_µ_g -77.840 9.928 -7.840 4.32e-13 ***
## Cortisol1 -83.545 9.928 -8.415 1.39e-14 ***
## Fe:Photons NA NA NA NA
## Fe:Gravitys_µ_g -131.183 18.126 -7.237 1.41e-11 ***
## Photons:Gravitys_µ_g -27.129 17.196 -1.578 0.116473
## Fe:Cortisol1 -151.521 17.196 -8.811 1.23e-15 ***
## Photons:Cortisol1 -16.394 17.196 -0.953 0.341744
## Gravitys_µ_g:Cortisol1 66.829 14.199 4.706 5.12e-06 ***
## Fe:Photons:Gravitys_µ_g NA NA NA NA
## Fe:Photons:Cortisol1 NA NA NA NA
## Fe:Gravitys_µ_g:Cortisol1 95.704 25.253 3.790 0.000208 ***
## Photons:Gravitys_µ_g:Cortisol1 20.804 24.411 0.852 0.395250
## Fe:Photons:Gravitys_µ_g:Cortisol1 NA NA NA NA
## ---
## Signif. codes: 0 '***' 0.001 '**' 0.01 '*' 0.05 '.' 0.1 ' ' 1
##
## Residual standard error: 34.39 on 174 degrees of freedom
## Multiple R-squared: 0.8449, Adjusted R-squared: 0.8351
## F-statistic: 86.18 on 11 and 174 DF, p-value: < 2.2e-16
##
## Analysis of Variance Table
##
## Response: Cytokine_Level
## Df Sum Sq Mean Sq F value Pr(>F)
## Fe 1 335708 335708 283.8161 < 2.2e-16 ***
## Photons 1 1406 1406 1.1883 0.2771703
## Gravity 1 220988 220988 186.8290 < 2.2e-16 ***
## Cortisol 1 292086 292086 246.9368 < 2.2e-16 ***
## Fe:Gravity 1 48789 48789 41.2473 1.236e-09 ***
## Photons:Gravity 1 1880 1880 1.5896 0.2090649
## Fe:Cortisol 1 99311 99311 83.9597 < 2.2e-16 ***
## Photons:Cortisol 1 219 219 0.1850 0.6676615
## Gravity:Cortisol 1 103704 103704 87.6737 < 2.2e-16 ***
## Fe:Gravity:Cortisol 1 16328 16328 13.8041 0.0002732 ***
## Photons:Gravity:Cortisol 1 859 859 0.7263 0.3952498
## Residuals 174 205814 1183
## ---
## Signif. codes: 0 '***' 0.001 '**' 0.01 '*' 0.05 '.' 0.1 ' ' 1
##
## ANOVA for IL_5
##
## Call:
## lm(formula = Cytokine_Level ~ Fe * Photons * Gravity * Cortisol,
## data = cytokine_data_no_outlier)
##
## Residuals:
## Min 1Q Median 3Q Max
## -73.347 -6.757 -0.157 4.982 105.141
##
## Coefficients: (4 not defined because of singularities)
## Estimate Std. Error t value Pr(>|t|)
## (Intercept) 100.000 4.170 23.981 < 2e-16 ***
## Fe 66.346 7.222 9.186 < 2e-16 ***
## Photons 4.478 7.222 0.620 0.536
## Gravitys_µ_g -82.438 5.897 -13.979 < 2e-16 ***
## Cortisol1 -83.766 5.961 -14.053 < 2e-16 ***
## Fe:Photons NA NA NA NA
## Fe:Gravitys_µ_g -7.312 10.214 -0.716 0.475
## Photons:Gravitys_µ_g -7.889 10.214 -0.772 0.441
## Fe:Cortisol1 -46.699 10.251 -4.555 9.74e-06 ***
## Photons:Cortisol1 -1.806 10.251 -0.176 0.860
## Gravitys_µ_g:Cortisol1 76.308 8.479 9.000 3.59e-16 ***
## Fe:Photons:Gravitys_µ_g NA NA NA NA
## Fe:Photons:Cortisol1 NA NA NA NA
## Fe:Gravitys_µ_g:Cortisol1 -6.983 14.634 -0.477 0.634
## Photons:Gravitys_µ_g:Cortisol1 5.785 14.526 0.398 0.691
## Fe:Photons:Gravitys_µ_g:Cortisol1 NA NA NA NA
## ---
## Signif. codes: 0 '***' 0.001 '**' 0.01 '*' 0.05 '.' 0.1 ' ' 1
##
## Residual standard error: 20.43 on 176 degrees of freedom
## Multiple R-squared: 0.8486, Adjusted R-squared: 0.8391
## F-statistic: 89.67 on 11 and 176 DF, p-value: < 2.2e-16
##
## Analysis of Variance Table
##
## Response: Cytokine_Level
## Df Sum Sq Mean Sq F value Pr(>F)
## Fe 1 50787 50787 121.7000 < 2.2e-16 ***
## Photons 1 3 3 0.0072 0.9324
## Gravity 1 109575 109575 262.5724 < 2.2e-16 ***
## Cortisol 1 159485 159485 382.1717 < 2.2e-16 ***
## Fe:Gravity 1 583 583 1.3963 0.2389
## Photons:Gravity 1 104 104 0.2485 0.6188
## Fe:Cortisol 1 22939 22939 54.9680 4.959e-12 ***
## Photons:Cortisol 1 18 18 0.0441 0.8340
## Gravity:Cortisol 1 67876 67876 162.6489 < 2.2e-16 ***
## Fe:Gravity:Cortisol 1 176 176 0.4228 0.5164
## Photons:Gravity:Cortisol 1 66 66 0.1586 0.6909
## Residuals 176 73447 417
## ---
## Signif. codes: 0 '***' 0.001 '**' 0.01 '*' 0.05 '.' 0.1 ' ' 1
##
## ANOVA for IL_13
##
## Call:
## lm(formula = Cytokine_Level ~ Fe * Photons * Gravity * Cortisol,
## data = cytokine_data_no_outlier)
##
## Residuals:
## Min 1Q Median 3Q Max
## -63.401 -6.955 0.000 8.212 68.074
##
## Coefficients: (4 not defined because of singularities)
## Estimate Std. Error t value Pr(>|t|)
## (Intercept) 100.000 3.601 27.768 < 2e-16 ***
## Fe 54.575 6.237 8.750 5.88e-15 ***
## Photons 3.035 6.237 0.487 0.6273
## Gravitys_µ_g -80.579 5.341 -15.086 < 2e-16 ***
## Cortisol1 -63.937 5.694 -11.229 < 2e-16 ***
## Fe:Photons NA NA NA NA
## Fe:Gravitys_µ_g -10.517 8.967 -1.173 0.2428
## Photons:Gravitys_µ_g 3.382 9.097 0.372 0.7106
## Fe:Cortisol1 -19.976 9.181 -2.176 0.0312 *
## Photons:Cortisol1 3.997 9.181 0.435 0.6640
## Gravitys_µ_g:Cortisol1 52.562 9.322 5.639 9.03e-08 ***
## Fe:Photons:Gravitys_µ_g NA NA NA NA
## Fe:Photons:Cortisol1 NA NA NA NA
## Fe:Gravitys_µ_g:Cortisol1 4.207 13.892 0.303 0.7625
## Photons:Gravitys_µ_g:Cortisol1 -7.813 16.456 -0.475 0.6357
## Fe:Photons:Gravitys_µ_g:Cortisol1 NA NA NA NA
## ---
## Signif. codes: 0 '***' 0.001 '**' 0.01 '*' 0.05 '.' 0.1 ' ' 1
##
## Residual standard error: 17.64 on 141 degrees of freedom
## Multiple R-squared: 0.8568, Adjusted R-squared: 0.8457
## F-statistic: 76.72 on 11 and 141 DF, p-value: < 2.2e-16
##
## Analysis of Variance Table
##
## Response: Cytokine_Level
## Df Sum Sq Mean Sq F value Pr(>F)
## Fe 1 29968 29968 96.2814 < 2.2e-16 ***
## Photons 1 396 396 1.2709 0.26150
## Gravity 1 123057 123057 395.3626 < 2.2e-16 ***
## Cortisol 1 85549 85549 274.8571 < 2.2e-16 ***
## Fe:Gravity 1 6 6 0.0197 0.88844
## Photons:Gravity 1 2 2 0.0071 0.93310
## Fe:Cortisol 1 912 912 2.9286 0.08922 .
## Photons:Cortisol 1 48 48 0.1557 0.69372
## Gravity:Cortisol 1 22583 22583 72.5560 2.211e-14 ***
## Fe:Gravity:Cortisol 1 85 85 0.2730 0.60217
## Photons:Gravity:Cortisol 1 70 70 0.2254 0.63569
## Residuals 141 43886 311
## ---
## Signif. codes: 0 '***' 0.001 '**' 0.01 '*' 0.05 '.' 0.1 ' ' 1
##
## ANOVA for IL_10
##
## Call:
## lm(formula = Cytokine_Level ~ Fe * Photons * Gravity * Cortisol,
## data = cytokine_data_no_outlier)
##
## Residuals:
## Min 1Q Median 3Q Max
## -88.351 -4.312 -1.018 0.000 173.884
##
## Coefficients: (4 not defined because of singularities)
## Estimate Std. Error t value Pr(>|t|)
## (Intercept) 100.000 4.411 22.671 < 2e-16 ***
## Fe 129.584 7.640 16.961 < 2e-16 ***
## Photons 4.787 7.868 0.608 0.544
## Gravitys_µ_g -96.764 6.238 -15.512 < 2e-16 ***
## Cortisol1 -75.930 6.238 -12.172 < 2e-16 ***
## Fe:Photons NA NA NA NA
## Fe:Gravitys_µ_g -119.091 10.805 -11.022 < 2e-16 ***
## Photons:Gravitys_µ_g -2.621 10.967 -0.239 0.811
## Fe:Cortisol1 -91.330 10.967 -8.328 2.19e-14 ***
## Photons:Cortisol1 3.372 10.967 0.307 0.759
## Gravitys_µ_g:Cortisol1 73.927 8.870 8.335 2.10e-14 ***
## Fe:Photons:Gravitys_µ_g NA NA NA NA
## Fe:Photons:Cortisol1 NA NA NA NA
## Fe:Gravitys_µ_g:Cortisol1 88.557 15.423 5.742 4.00e-08 ***
## Photons:Gravitys_µ_g:Cortisol1 -4.163 15.423 -0.270 0.788
## Fe:Photons:Gravitys_µ_g:Cortisol1 NA NA NA NA
## ---
## Signif. codes: 0 '***' 0.001 '**' 0.01 '*' 0.05 '.' 0.1 ' ' 1
##
## Residual standard error: 21.61 on 177 degrees of freedom
## Multiple R-squared: 0.893, Adjusted R-squared: 0.8864
## F-statistic: 134.4 on 11 and 177 DF, p-value: < 2.2e-16
##
## Analysis of Variance Table
##
## Response: Cytokine_Level
## Df Sum Sq Mean Sq F value Pr(>F)
## Fe 1 73947 73947 158.3563 < 2.2e-16 ***
## Photons 1 172 172 0.3680 0.5449
## Gravity 1 302858 302858 648.5621 < 2.2e-16 ***
## Cortisol 1 119998 119998 256.9724 < 2.2e-16 ***
## Fe:Gravity 1 50463 50463 108.0656 < 2.2e-16 ***
## Photons:Gravity 1 108 108 0.2304 0.6318
## Fe:Cortisol 1 18393 18393 39.3882 2.597e-09 ***
## Photons:Cortisol 1 48 48 0.1021 0.7498
## Gravity:Cortisol 1 106297 106297 227.6320 < 2.2e-16 ***
## Fe:Gravity:Cortisol 1 17828 17828 38.1791 4.316e-09 ***
## Photons:Gravity:Cortisol 1 34 34 0.0729 0.7875
## Residuals 177 82653 467
## ---
## Signif. codes: 0 '***' 0.001 '**' 0.01 '*' 0.05 '.' 0.1 ' ' 1

# Print AIC values for each cytokine
print(aic_values)

## IFN_G
## 0.000 0.000 0.000 0.000 0.000 0.000 0.000 1747.755
## TNF_A IL_2 IL_4 IL_5 IL_13 IL_10
## 1761.283 1842.666 1857.516 1681.482 1326.010 1711.604

View(aic_values)

vif(lm_model_noout, type = "predictor")

## Warning in cor(X): the standard deviation is zero

## GVIFs computed for predictors

## GVIF Df GVIF^(1/(2*Df)) Interacts With Other Predictors
## Fe 1 15 1 Photons, Gravity, Cortisol --
## Photons 1 15 1 Fe, Gravity, Cortisol --
## Gravity 1 15 1 Fe, Photons, Cortisol --
## Cortisol 1 15 1 Fe, Photons, Gravity --

## Flow cytometry data analysis

The cells were stained for intracellular cytokines to evaluate the production of the characteristic Th cytokines (IFN-γ, TNF-α, IL-2, IL-4, and IL-10). Results show the normalized mean fluorescence intensity (MFI) normalized to the control condition. We observe a considerable variability between donors, particularly prominent for IFN-γ, IL-2 and IL-10 (Figure 4, A, C, and D).

For TNF-α we see that for the combined exposures with Fe ions is likely to induce an increase in the MFI, compared to the other conditions.

# Supplementary Figures and Tables

## Supplementary Figures


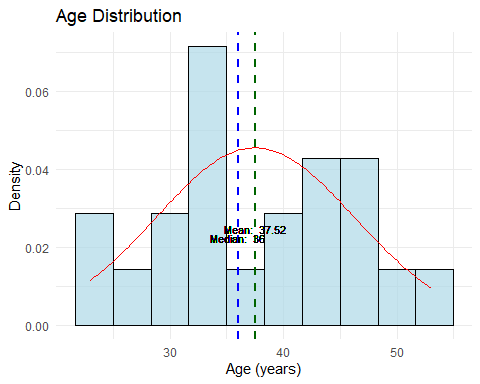


**Figure 1. Histogram of the age distribution of the study population**


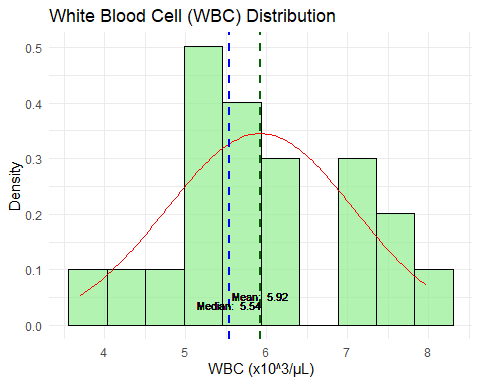


Figure 2. Histogram of the white blood cell count (WBC) distribution in the population


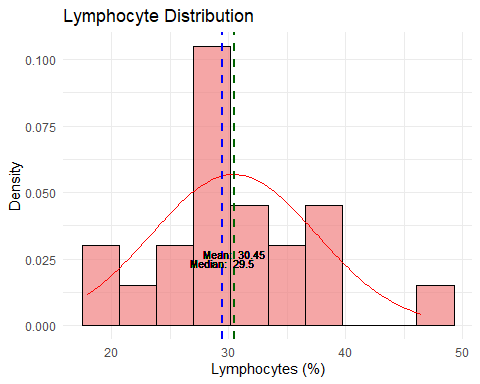


Figure 3. Distribution of the lymphocyte percentage in the study population


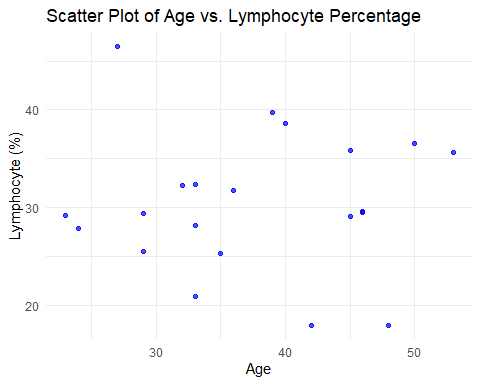


Figure 4. Correlation analysis of the age and lymphocyte percentage in the study population


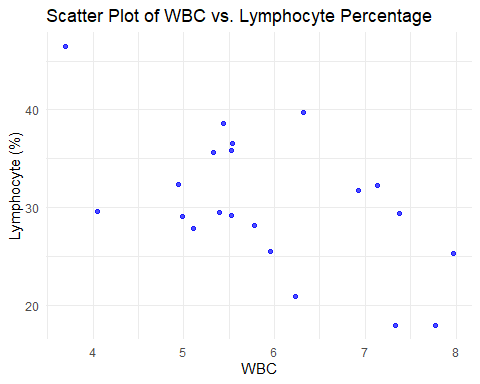


Figure 5. Correlation analysis of the WBC and the lymphocyte percentage


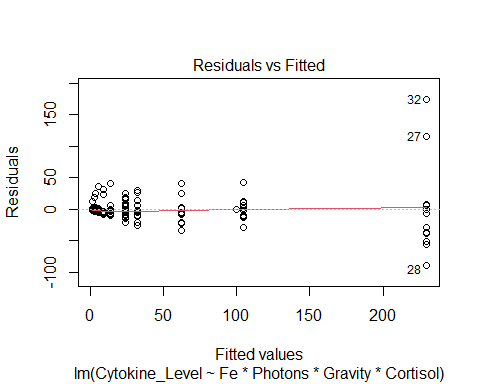


Figure 6. Visual inspection of the fitness of the linear regression model – Residuals vs Fitted


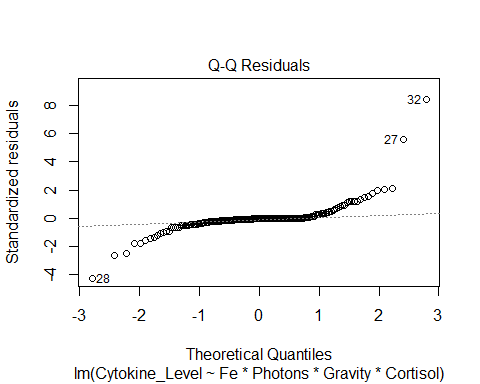


Figure 7. Visual inspection of the fitness of the linear regression model – Q-Q residuals


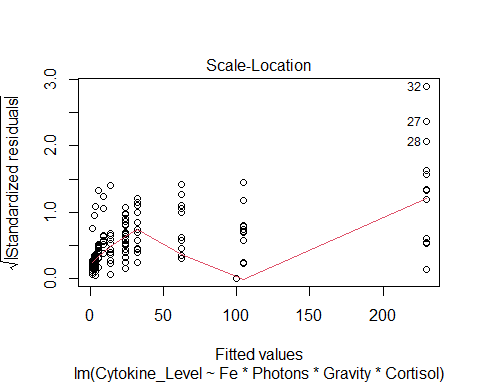


**Figure 8. Visual inspection of the fitness of the linear regression model – Scale-location**


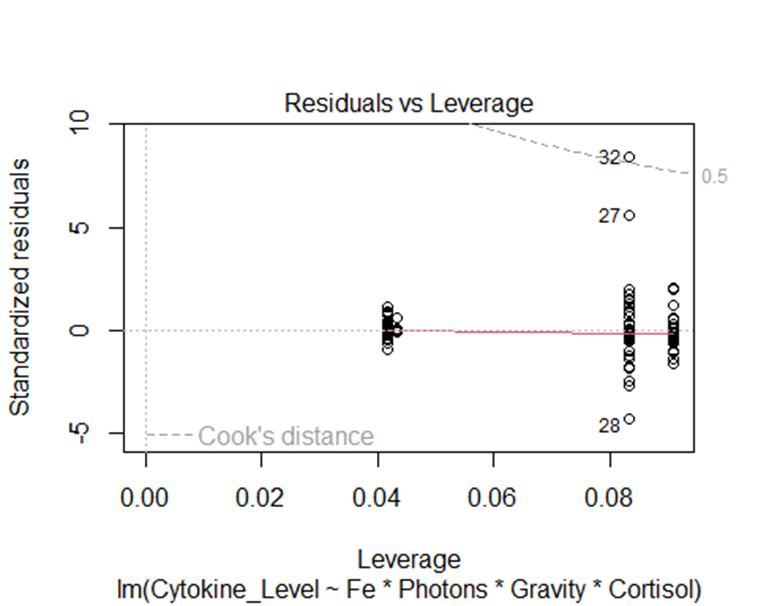


**Figure 9. Visual inspection of the fitness of the linear regression model – Residuals vs Leverage**

**
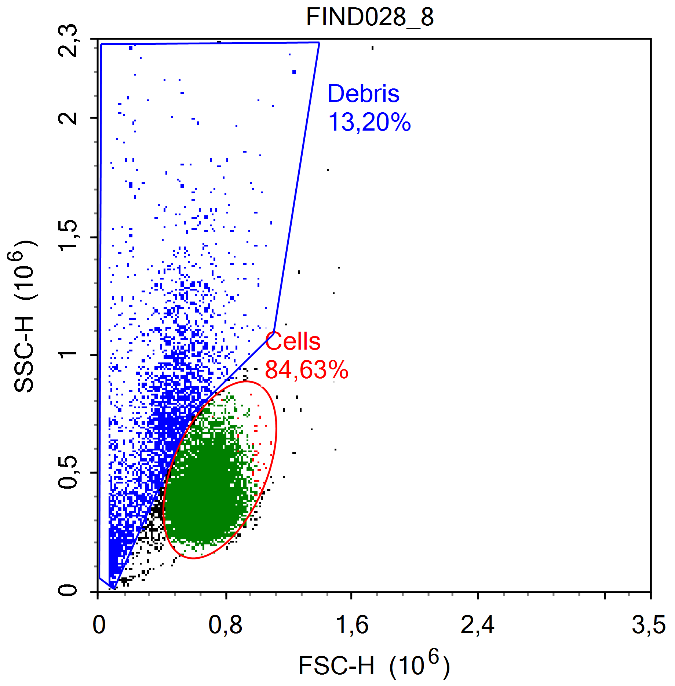

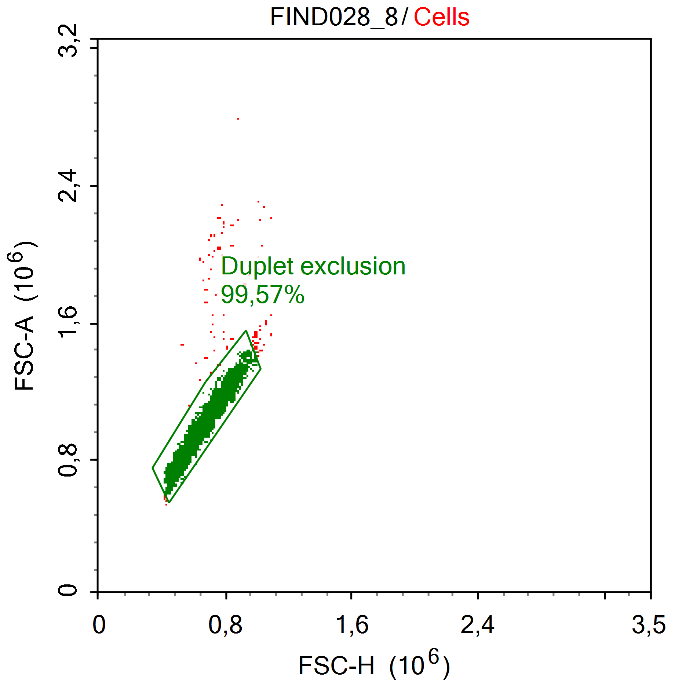

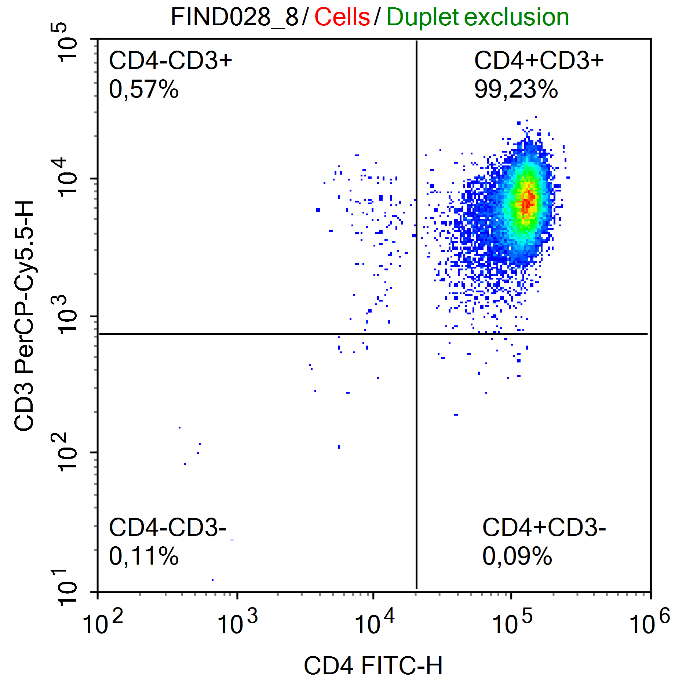
**

Figure 10 – Flow cytometry gating strategy for CD4+ T cell population. Data shown for donor Number FIND028

*
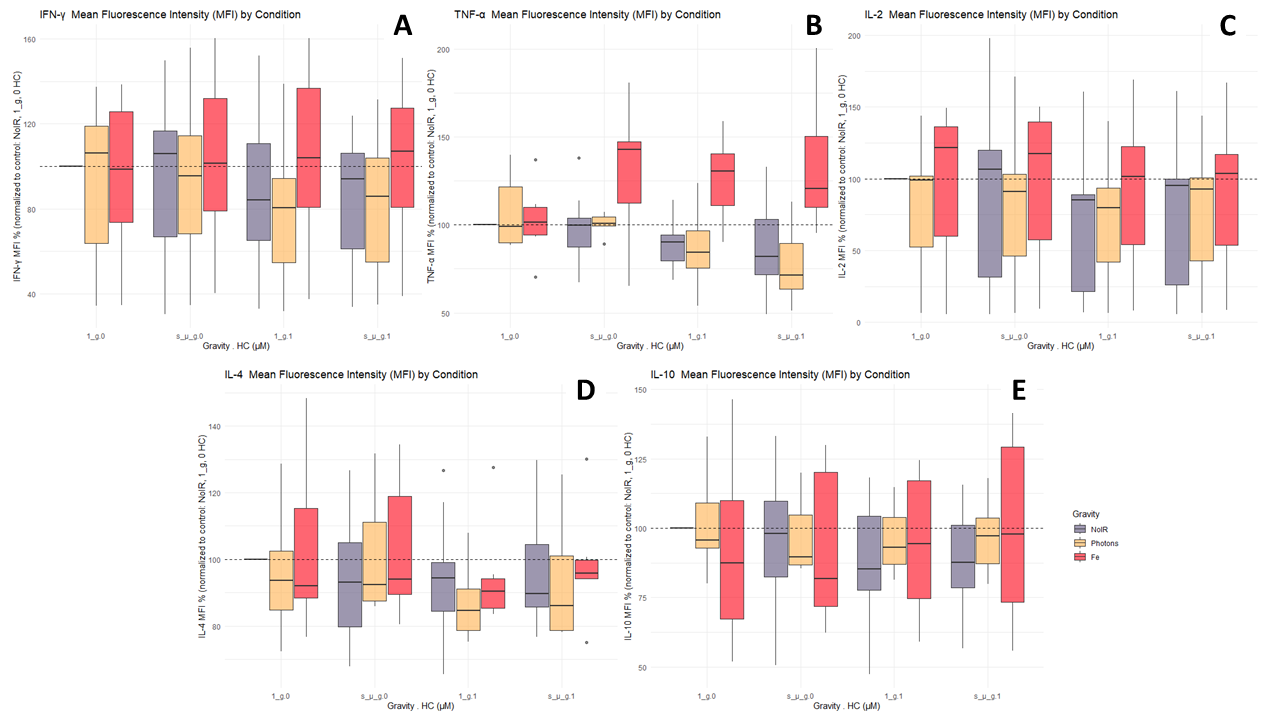
*

Figure 11. CD4+ T cell cytokines measured in flow cytometry. A: IFN-γ; B: TNF-α; C: IL-2; D: IL-4; E: IL-10. 1_g: normal gravity; s_µ_g: Simulated microgravity; HC: Hydrocortisone. Values represented are normalized to the control (No Ionizing radiation (IR), 0 µM HC, 1_g). Plot shows boxplot with median as center line. The bottom and top edges of the box represent the 1st quartile (Q1) and the 3rd quartile (Q3) of the data, respectively. The height of the box (the interquartile range or IQR) represents the middle 50% of the data. The whiskers represent 1.5 times the IQR. Points represent outliers. The x axis shows the gravity levels combined with HC. comparing the distribution of the normalized cytokine levels across different IR exposures (No IR, Photons, Fe (ions)), represented by different fill colors of the boxes. The horizontal dashed line is indicating the average value for the control condition. Data was obtained from 6 donors and analyzed with RStudio v4.3.1.
